# A Multi-Omics Study Reveals the Active Components and Therapeutic Mechanism of Erhuang Quzhi Formula for Non-Alcoholic Fatty Liver Disease

**DOI:** 10.3390/nu17243849

**Published:** 2025-12-10

**Authors:** Teng Ma, Mingzhu Li, Yuan Liu, Yu Chen, Zipeng Guan, Tonghua Liu, Dongmei Qin, Jia Xu

**Affiliations:** 1 Key Laboratory of Health Cultivation of Traditional Chinese Medicine, The Ministry of Education, The Third Clinical Medical College, Beijing University of Chinese Medicine, Beijing 100029, China; mt136@bucm.edu.cn (T.M.); liuyuan@bucm.edu.cn (Y.L.); 20250941394@bucm.edu.cn (Y.C.); 13321991850@163.com (Z.G.); 2 State Key Laboratory of Traditional Chinese Medicine Syndrome and Guangdong Provincial Key Laboratory of Syndrome and Formula, School of Pharmaceutical Sciences, Guangzhou University of Chinese Medicine, Guangzhou 510006, China; 20251112228@stu.gzucm.edu.cn; 3 Chinese Medicine Guangdong Laboratory (Hengqin Laboratory), Guangdong-Macao In-Depth Cooperation Zone in Hengqin, Hengqin, Zhuhai 519000, China; 4 Key Laboratory of Health Cultivation of Traditional Chinese Medicine, The Ministry of Education, Beijing University of Chinese Medicine, Beijing 100029, China; thliu@vip.163.com; 5 Key Laboratory of Xinjiang Phytomedicine Resource and Utilization, Ministry of Education, School of Pharmacy, Shihezi University, Shihezi 832003, China

**Keywords:** non-alcoholic fatty liver disease, Erhuang Quzhi Formula, serum pharmacochemistry, lipidomics, metabolomics

## Abstract

**Objectives**: Erhuang Quzhi Formula (EQF) has been used for the treatment of non-alcoholic fatty liver disease (NAFLD). However, its active components and mechanistic basis remain unclear. This study aims to systematically identify the therapeutic material basis of EQF and to elucidate its potential mechanisms of action against NAFLD through an integrated multi-omics strategy. **Methods**: An integrated strategy combining UPLC-Q-TOF-MS and network pharmacology was applied to characterize serum components of EQF and construct a compound–target network. Core targets were screened and validated by molecular docking. A NAFLD model was established in C57BL/6 mice through high-fat diet feeding. To evaluate the therapeutic effects, mice were treated with EQF and assessed by measurements of serum biochemical parameters, liver histopathology, and glucose tolerance. UPLC-Q-TOF-based lipidomic and metabolomic analyses of liver tissue were conducted to clarify EQF’s regulatory effects on global lipid profiles and endogenous metabolites. Key genes and proteins involved in relevant signaling pathways were verified by RT-qPCR and Western blot. **Results**: A total of thirty-one prototype compounds were identified in the EQF-containing serum. Network pharmacology analysis predicted that EQF may alleviate NAFLD by acting on core targets such as TNF, JUN, and STAT3. In vivo experiments demonstrated that EQF administration significantly improved liver function, attenuated dyslipidemia, and reduced inflammation in model mice. Furthermore, metabolomic and lipidomic analyses indicated that EQF effectively reversed abnormal glycerophospholipid and sphingolipid levels and restored their metabolic homeostasis. **Conclusions**: EQF exerts therapeutic effects in a NAFLD mouse model through multi-component, multi-target, and multi-pathway mechanisms, primarily associated with the regulation of lipid metabolism, improvement of liver function, and suppression of inflammatory responses. This study provides mechanistic insights and a pharmacodynamic basis for the future clinical investigation of EQF.

## 1. Introduction

Non-alcoholic fatty liver disease (NAFLD) is a metabolically associated liver injury driven by insulin resistance and genetic susceptibility [1]. Its defining pathological feature is the excessive accumulation of triglycerides within hepatocytes, known as hepatic steatosis, which is diagnosed after excluding other established causes of liver damage such as significant alcohol consumption [2]. As the global prevalence of obesity and type 2 diabetes continues to rise, NAFLD has become one of the most common chronic liver diseases worldwide, affecting approximately one-quarter of the global population and imposing a substantial burden on public health systems [3]. NAFLD encompasses a broad disease spectrum, which may progress from simple steatosis to non-alcoholic steatohepatitis (NASH). The latter is characterized not only by lipid accumulation, but also by hepatocyte ballooning, lobular inflammation, and varying degrees of fibrosis [4,5]. NASH represents a critical stage in disease progression, as it can lead to liver fibrosis, cirrhosis, and even hepatocellular carcinoma, posing serious risks to patient survival [6]. Although the pathogenesis of NAFLD remains incompletely understood, the earlier “two-hit hypothesis” has been largely superseded by the “multiple parallel hits” theory [7]. This updated model emphasizes the synergistic roles of insulin resistance, disordered lipid metabolism, gut microbiota dysbiosis, innate immune activation, and genetic factors in driving disease initiation and progression [8,9]. Currently, there are no approved pharmacotherapies specifically for NASH. Clinical management continues to rely primarily on lifestyle interventions—including dietary modification and increased physical activity—aimed at weight reduction, complemented by medications that target associated metabolic abnormalities, such as dyslipidemia and insulin resistance [10]. Given the complex and multifactorial nature of NAFLD, elucidating its underlying mechanisms and developing effective treatments remain the main priorities and challenges in hepatology research.

Traditional Chinese medicine (TCM) demonstrates distinctive advantages and considerable potential in the prevention and management of metabolic disorders. As a clinically validated empirical formula, the Erhuang Quzhi Formula (EQF) traces its origins to the “Liver-Protecting and Toxin-Suppressing Formulas” documented in *Yuan Jinqi’s Medical Essays* by the esteemed TCM specialist Professor Yuan Jinqi [11]. The formulation consists of thirteen herbal components, including *Rhei Radix* et Rhizoma (Dahuang), *Curcumae Longae* Rhizoma (Jianghuang), and *Glycyrrhizae Radix* et Rhizoma (Gancao) [12]. Clinical evidence supports the efficacy of EQF for NAFLD treatment, demonstrating improvements in clinical symptoms, dyslipidemia, and hepatic function, along with a favorable safety profile [13]. Mechanistic investigations have elucidated that EQF exerts its effects through dual regulatory pathways [14]: it suppresses MAPK-mediated inflammatory and apoptotic signaling while potentiating AKT/STAT3-dependent survival pathways, collectively contributing to reduced lipogenesis, attenuated inflammation, and enhanced hepatoprotection. Furthermore, EQF upregulates uncoupling protein 1 (UCP1) expression, stimulating white adipose tissue browning and activating brown adipose tissue thermogenesis. These processes collectively enhance systemic energy expenditure, alleviate hepatic steatosis, and promote metabolic homeostasis [15]. Nevertheless, the comprehensive characterization of EQF’s pharmacologically active constituents and their intricate mechanisms of action remains incomplete due to the formula’s complex chemical profile. This knowledge gap substantially impedes its contemporary progress and international recognition.

Serum pharmacochemistry, lipidomics, and metabolomics represent crucial technological approaches for systematically elucidating the pharmacodynamic material basis and mechanistic actions of TCM compound formulations [16,17]. Serum pharmacochemistry focuses on analyzing blood-absorbed components, enabling the direct screening of potential bioactive constituents that mediate TCM’s therapeutic effects in vivo [18]. Lipidomics provides a comprehensive methodology for the systematic profiling of lipid molecules within biological systems [19], demonstrating particular relevance for investigating metabolic disorders, such as NAFLD [20]. This approach precisely characterizes pathological alterations in hepatic lipid metabolic profiles and sensitively detects drug-induced modulations of key lipid species, including triglycerides and cholesteryl esters, thereby uncovering the molecular targets of pharmacological interventions [21]. Complementarily, metabolomics conducts global analyses of endogenous low-molecular-weight metabolites (e.g., amino acids and bile acids), capturing dynamic fluctuations that reflect the organism’s overall metabolic status [22]. Through the comparative assessment of metabolic pathway perturbations before and after treatment, this strategy enables the holistic evaluation of a drug’s capacity to restore metabolic homeostasis from a systems biology perspective [23,24]. The integration of these three methodologies establishes a complete investigative pipeline spanning from “identification of bioactive substances in circulation” to the “resolution of specific metabolic targets,” and ultimately to an “assessment of integrated physiological outcomes [25,26].” This multi-omics framework provides powerful support for comprehensively deciphering the modern pharmacological mechanisms underlying TCM compound therapies.

This study integrates serum pharmacochemistry, lipidomics, and metabolomics to establish a multidimensional research framework for systematically elucidating the therapeutic mechanism of EQF against NAFLD. Specifically, serum pharmacochemistry will identify bioactive constituents absorbed into circulation to clarify the material basis of efficacy; lipidomics will characterize the formula’s regulation of hepatic lipid metabolic networks; and metabolomics will elucidate the systemic mechanisms through which EQF restores metabolic homeostasis. This integrated multi-omics strategy will provide compelling evidence for deciphering EQF’s mode of action and establish a new paradigm for modernizing research on TCM compound formulations.

## 2. Materials and Methods

### 2.1. Chemical and Reagents

Erhuang Quzhi Formula comprises 13 herbal components: *Curcumae Longae* Rhizoma, *Rhei Radix* et Rhizoma, *Atractylodis Macrocephalae* Rhizoma, *Puerariae Lobatae* Radix, *Nelumbinis Folium*, *Alismatis* Rhizoma, *Salviae Miltiorrhizae Radix* et Rhizoma, *Polygoni Cuspidati Rhizoma* et Radix, *Hirudo*, *Bombyx Batryticatus*, *Lapis Micae*, *Gynostemmatis Herba*, and *Glycyrrhizae Radix* et Rhizoma. These herbs were supplied by the pharmacy of the Third Affiliated Hospital of Beijing University of Chinese Medicine.

Fenofibrate (Feno) capsules (Laboratoires Fournier, Paris, France) served as the positive control. Experimental diets consisted of standard feed and high-fat feed (60% fat, Xietong Biotechnology Co., Ltd., Nanjing, China). The hematoxylin–eosin staining kit (BH0001) and Oil Red O staining solution (B0023) were obtained from Wuhan Boerfu Biotechnology Co., Ltd. (Wuhan, China). Mass-spectrometry-grade acetonitrile, methanol, and formic acid were sourced from CNW Technologies (Shanghai, China).

### 2.2. Preparation of EQF Decoction

The herbal formula was prepared as follows: *Lapis Micae* (30 g), *Puerariae Lobatae* Radix (20 g), *Atractylodis Macrocephalae* Rhizoma (15 g), *Salviae Miltiorrhizae Radix* et Rhizoma (15 g), *Gynostemmatis Herba* (15 g), *Polygoni Cuspidati Rhizoma* et Radix (15 g), *Curcumae Longae* Rhizoma (12 g), *Alismatis* Rhizoma (12 g), *Bombyx Batryticatus* (10 g), *Nelumbinis Folium* (10 g), *Rhei Radix* et Rhizoma (9 g), *Glycyrrhizae Radix* et Rhizoma (6 g), and *Hirudo* (3 g). The herbs were soaked in 10 volumes of distilled water for 30 min, boiled vigorously and then simmered for 30 min, and filtered. The residue was re-extracted with 8 volumes of water by simmering for another 30 min and filtered. The combined filtrates were concentrated to 1 g/mL (in terms of raw herb weight), aliquoted, and stored at 4 °C.

### 2.3. Preparation of EQF-Containing Serum

Male Sprague-Dawley rats (SPF grade, 200 ± 20 g, 6 weeks old) were supplied by Shanghai Jihui Laboratory Animal Co., Ltd. (Shanghai, China). Animals were housed under SPF conditions (22–25 °C, 55–60% humidity, 12 h light/dark cycle) with four rats per cage. They had free access to water and standard chow. All procedures were approved by the Institutional Animal Care and Use Committee of Shanghai Wuchuang Biotechnology (Shanghai) Co., Ltd. (approval no. WTPZ20250220001) and followed relevant animal welfare guidelines.

After one week of acclimatization, six rats with similar body weights were randomly assigned to two groups (*n* = 3 per group): control and EQF. The EQF group received EQF decoction (15.12 g·kg^−1^·d^−1^) by oral gavage for 7 days. The decoction was suspended in distilled water. Control rats received an equal volume of solvent.

Blood was collected from the retro-orbital plexus under isoflurane anesthesia before dosing (blank serum) and at 0.5, 1, and 2 h after the last administration. Serum was separated by centrifugation. Equal volumes from the three post-dose time points were pooled to prepare EQF-containing serum.

Aliquots (4 mL) of blank serum and EQF-containing serum were mixed with three volumes of cold MS-grade methanol. After vortexing and incubation at 4 °C, samples were centrifuged. The supernatant was collected, dried, and stored at −80 °C. For analysis, the residue was reconstituted in 50% methanol, centrifuged, and the supernatant was used.

### 2.4. Analysis of EQF Decoction and EQF-Containing Serum Components

The chemical compositions of EQF decoction, blank serum and EQF-containing serum were analyzed by UPLC-Q-TOF-MS. Chromatographic separation was achieved on a Welch Ultimate AQ-C18 (Welch Equipment Co., Ltd., Shanghai, China) column (2.1 × 100 mm, 1.8 µm) held at 40 °C, using a gradient of 0.1% formic acid in water (A) and acetonitrile (B) (97% to 100% B from 0 to 48 min) at 0.3 mL/min. The injection volume was 5 µL. Mass detection was performed in ESI positive and negative modes with scan ranges *m*/*z* 50–1700 (MS) and *m*/*z* 50–1250 (MS/MS). The ion source temperature was 500 °C, the collision energy was 10 eV, and the declustering potential was 100 V. Data acquisition used Analyst TF 1.7.1, and processing was performed with Peak View 1.2. During identification, mass spectrometry data was matched with the 1.0 database of Natural Products HR-MS/MS Spectral Library, and the compounds were preliminarily screened according to the scores of each chromatographic peak, and further confirmed according to the primary and secondary information of each chromatographic peak and the related literature.

### 2.5. Screening of Active Components and Targets of EQF Against NAFLD

A total of 31 prototype components were identified in the drug-containing rat serum. Their chemical structures and related information were retrieved from the PubChem (https://pubchem.ncbi.nlm.nih.gov/ accessed on 30 July 2025) and SciFinder-n (https://scifinder-n.cas.org/ accessed on 30 July 2025) databases. Protein targets of these components were predicted using the Swiss Target Prediction platform. Disease targets associated with nonalcoholic fatty liver disease (NAFLD) were collected from the GeneCards (https://www.genecards.org/ accessed on 30 July 2025), OMIM (https://www.omim.org/ accessed on 30 July 2025), and DisGENET (https://www.disgenet.org/ accessed on 30 July 2025) databases. Potential therapeutic targets of EQF for NAFLD were identified by intersecting the predicted compound targets with the NAFLD-related targets.

### 2.6. Protein–Protein Interaction (PPI) Network Construction

The protein–protein interaction (PPI) data were acquired from the STRING database (https://string-db.org/ accessed on 30 July 2025) using Homo sapiens as the species and a minimum interaction confidence score of 0.4. The interaction data were then imported into Cytoscape (v3.10.3) for PPI network construction and visualization. Network topology was analyzed using the CentiScaPe (v2.2) plug-in. Hub targets were identified based on three calculated centrality parameters for each node: betweenness centrality, closeness centrality, and degree centrality.

### 2.7. Molecular Docking

The 3D structures of six compounds (aloe-emodin, rhein, daidzein, nuciferin, liquiritin, and uralsaponin) were obtained from the PubChem database and prepared using Open Babel. The crystal structures of seven target proteins (BCL2, CASP3, ESR1, JUN, MMP9, STAT3, and TNF) were downloaded from the RCSB Protein Data Bank. Molecular docking was performed using AutoDockTools (v1.5.6) and AutoDock Vina (v1.1.2).

### 2.8. Animal Experiment

Male C57BL/6N mice (SPF grade, 6 weeks old, 19–23 g) were provided by Beijing Vital River Laboratory Animal Technology (SCXK: 2021-0006). The animals were housed under specific pathogen-free conditions (22–25 °C, 55–60% humidity, 12 h light/dark cycle) with four mice per cage, and allowed free access to water and a standard diet. All experimental procedures were approved by the Animal Welfare Ethics Committee of Beijing Zhongshan Jinqiao Experimental Animal Technology Development Co., Ltd. (Beijing, China) (approval no. 20240525YZE-3R) and conducted in accordance with relevant animal welfare guidelines.

Fifty mice were stratified by body weight and randomly assigned to two dietary regimens: a control group (*n* = 10) received a standard diet, while the remaining forty mice were fed a high-fat diet (HFD) for 9 weeks to induce non-alcoholic fatty liver disease (NAFLD). Mice that completed the 9-week HFD feeding and exhibited significant weight gain compared to controls were included in the study. No animals were excluded due to mortality or severe unrelated illness.

The final experimental groups (each *n* = 10) were as follows: control, model, low-dose EQF (EQF-L, 11.18 g·kg^−1^·d^−1^), high-dose EQF (EQF-H, 22.36 g·kg^−1^·d^−1^), and fenofibrate (Feno, 0.026 g·kg^−1^·d^−1^). The EQF was suspended in distilled water, and the fenofibrate was suspended in 1% CMC-Na solution. The control and model groups received an equal volume of solvent. The sample size was determined based on previous NAFLD studies [27,28,29].

Starting from week 10, the EQF-L, EQF-H, and Feno groups were administered the corresponding treatments by oral gavage daily for 8 weeks. The control and model groups received an equal volume of distilled water. Body weight was measured every week. At week 17, an oral glucose tolerance test (OGTT) was conducted after 12 h of fasting. Blood glucose levels were determined at 0, 30, 60, and 120 min following oral administration of glucose (2 g/kg). All experimental procedures were performed by trained staff to reduce animal discomfort. Humane endpoints were predefined as more than 20% unexplained body weight loss, severe distress, or inability to eat or drink. Animals were checked daily; no animal met the humane endpoint criteria or exhibited adverse events during the study.

### 2.9. Biochemical Analysis

All biochemical analyses were conducted by technicians blinded to the experimental group assignments to minimize observer bias. Serum samples were collected from the retro-orbital venous plexus after the final drug intervention and high-fat-diet feeding. The blood samples were allowed to stand at room temperature, followed by centrifugation at 3000 rpm for 15 min at 4 °C using a GTR10-1 high-speed refrigerated centrifuge (Times Beili, Beijing, China) to obtain serum. Serum levels of glucose (GLU), total cholesterol (TC), triglycerides (TG), low-density lipoprotein cholesterol (LDL-C), high-density lipoprotein cholesterol (HDL-C), alanine aminotransferase (ALT), aspartate aminotransferase (AST), and alkaline phosphatase (ALP) were measured using a BS-240Vet automatic biochemical analyzer (Mindray Animal Medical, Shenzhen, China). All biochemical analyses were performed by technicians blinded to group assignments to minimize bias.

### 2.10. Histological Staining

All histological assessments were conducted by technicians blinded to the experimental group assignments to minimize observer bias. At the end of the 17-week experiment, mice were anesthetized with 4% isoflurane in oxygen using an induction chamber. After loss of pedal reflexes, anesthesia was maintained with 1.5–2% isoflurane delivered via a nose cone, followed by euthanasia. Liver tissues were promptly collected for histological analysis. Histological scoring was conducted by technicians in a blinded manner to group assignments.

Hematoxylin and eosin (H&E) staining: liver tissues were fixed in 4% paraformaldehyde, embedded in paraffin, and sectioned at 4 μm. After dewaxing and rehydration, sections were stained with hematoxylin and eosin, followed by dehydration and mounting for morphological examination under a light microscope.

Oil Red O staining: fresh liver tissues were sectioned at 8 μm using a cryostat. After fixation in 4% paraformaldehyde and rinsing, sections were stained with an Oil Red O working solution (15 min, dark) and counterstained with hematoxylin. Lipid deposition was visualized under a light microscope.

### 2.11. Metabolomic Analysis

Mouse liver tissues were collected at the end of the experiment for metabolomic analysis by using a UPLC-Q-TOF-MS system (Waters Acquity I-Class PLUS coupled with Xevo G2-XS QTof, Milford, MA, USA). Chromatographic separation was achieved on an Acquity UPLC HSS T3 column (Waters, Milford, MA, USA) (1.8 μm, 2.1 × 100 mm) with a mobile phase consisting of 0.1% formic acid in water (A) and 0.1% formic acid in acetonitrile (B). The gradient elution program was set as follows: 0–0.5 min, 95% A; 0.5–5.5 min, 50% A; 5.5–10.5 min, 5% A; 10.5–12 min, 95% A. The flow rate was 400 μL/min and the injection volume was 2 μL. Mass spectrometric data were acquired using MassLynx V4.2. Peak extraction and alignment were processed with Progenesis QI, and metabolite identification was conducted by querying the METLIN database and a custom-built library.

### 2.12. Lipidomic Analysis

Lipidomic profiling was carried out using a UPLC-Q-TOF-MS system (Waters Acquity I-Class PLUS coupled with Xevo G2-XS QTof). Separation was performed on an Acquity UPLC CSH C18 column (1.8 μm, 2.1 × 100 mm). The mobile phase consisted of 60% acetonitrile with 10 mM ammonium acetate and 0.1% formic acid (A), and 90% isopropanol/acetonitrile with 10 mM ammonium acetate and 0.1% formic acid (B). The gradient elution was programmed as follows: 0–2 min, 60% A; 2–2.1 min, 57% A; 2.1–12 min, 50% A; 12–12.1 min, 46% A; 12.1–18 min, 30% A; 18–18.1 min, 1% A; 18.1–20 min, 60% A. The flow rate was maintained at 0.4 mL/min with an injection volume of 5 μL. Data acquisition was performed using MassLynx V4.2, and raw data were processed with Progenesis QI. Lipid identification was based on the METLIN database and a custom library, with a fragment mass tolerance of less than 100 ppm.

### 2.13. Real-Time Quantitative PCR Analysis

Total RNA was extracted from mouse liver tissues using TRIzol reagent (B511311-0100, Sangon Biotech, Shanghai, China), and its concentration and purity were determined. According to the MightyScript First Strand cDNA Synthesis Master Mix (B639251-0100, Sangon Biotech), 1 μg of total RNA was reverse-transcribed into cDNA. Quantitative PCR was performed using SYBR Green master mix (RK21203, ABconal, Wuhan, China) on a real-time PCR system under the following conditions: 95 °C for 5 min; 40 cycles of 95 °C for 15 s and 60 °C for 30 s. The relative mRNA expression of target genes was normalized to Hprt and calculated using the 2^^−ΔΔCt^ method. All reactions were run in triplicate with three independent replicates. Primer sequences are listed in Table S1.

### 2.14. Western Blot Analysis

The total protein was extracted from mouse liver tissues, and its concentration was quantified using a BCA assay (P0010S, Beyotime, Shanghai China). After separation by SDS-PAGE, proteins were transferred onto PVDF membranes. The blot were blocked with 5% skim milk in TBST for 1 h and subsequently incubated with the following primary antibodies: BCL-2 (1:1000, A19693, ABclonal, Wuhan, China), STAT3 Rabbit pAb (1:1000, A1192, ABclonal, Wuhan, China) Phospho-STAT3-Y705 (1:800, AP0070, ABclonal,, Wuhan, China), c-Jun (1:2000, A27455, ABclonal, Wuhan, China). Following incubation with HRP-conjugated Goat anti-Rabbit IgG (H+L) (1:5000, AS014, ABclonal, Wuhan, China) for 1 h, blots were visualized by chemiluminescence, and band intensities were analyzed with ImageJ2 using β-actin as the loading control.

### 2.15. Statistical Analysis

All data are expressed as mean ± SD. Statistical analyses were performed using GraphPad Prism 10.0 software. The normality of data distribution was assessed using the Shapiro–Wilk test, and homogeneity of variances was evaluated using the Brown–Forsythe test. For multi-group comparisons, one-way ANOVA with Tukey’s post hoc test or the Kruskal–Wallis test with Dunn’s post hoc test were applied based on the results of normality and variance tests. Comparisons between the two groups were performed using the unpaired *t*-test or the Mann–Whitney U test. A *p*-value less than 0.05 was considered statistically significant.

## 3. Results

### 3.1. Identification of EQF Components and Serum Absorption Components by UPLC-Q-TOF-MS

**Figure 1:**
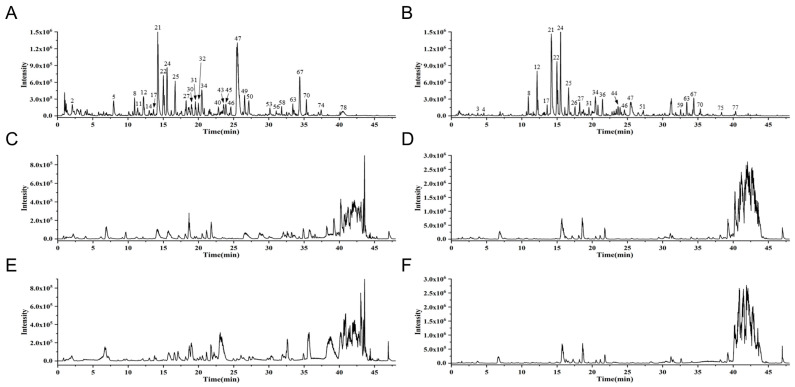
UPLC-Q-TOF-MS base peak chromatograms (BPC) of EQF decoction, blank serum, and EQF-containing serum samples. (**A**) EQF decoction in negative-ion mode. (**B**) EQF decoction in positive-ion mode. (**C**) Blank serum in negative-ion mode. (**D**) Blank serum in positive-ion mode. (**E**) Serum sample collected after oral administration of EQF to rats in negative-ion mode. (**F**) Serum sample collected after oral administration of EQF to rats in positive-ion mode.

The chemical composition of EQF decoction, blank serum, and EQF-containing serum was analyzed by UPLC-Q-TOF-MS in both positive- and negative-ion modes. Representative base peak intensity chromatograms are shown in Figure 1. A total of 83 compounds were identified in the EQF decoction. In the EQF-containing serum, 106 compounds were detected. These included 31 prototype components derived from the decoction and 75 metabolites. Detailed retention times and mass data are provided in Table 1 and Table 2. The identified compounds in serum were categorized as 51 flavonoids, 30 quinones, 11 alkaloids, 8 phenolic acids, 4 triterpenoid saponins, and 2 triterpenes. Multiple metabolic reactions were observed, including glucuronidation, sulfation, methylation, hydroxylation, and hydrogenation. All detected ions exhibited high mass accuracy, with errors below 8 ppm. The chemical structures of the 31 prototype compounds are presented in Figure S1.

### 3.2. Network Pharmacology Analysis and Verification of EQF in the Treatment of NAFLD

**Figure 2:**
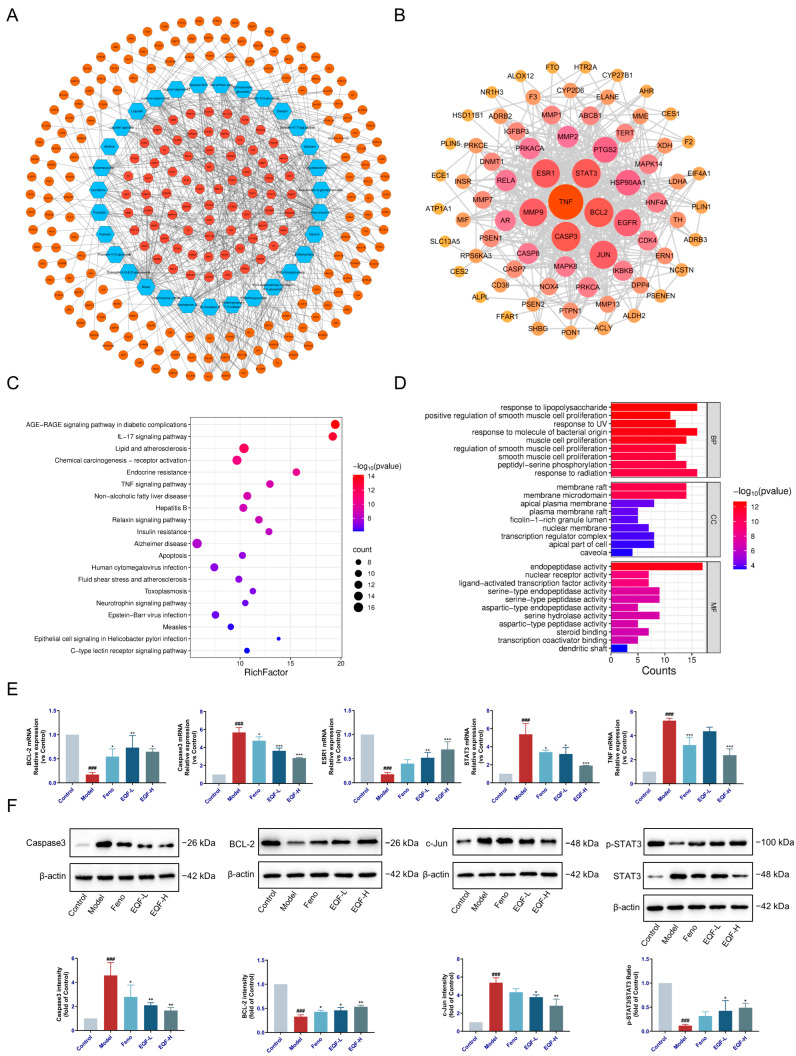
Network pharmacology analysis. (**A**) Compound–target network of EQF against NAFLD. Blue nodes represent chemical components of EQF; orange nodes denote potential targets. Light orange nodes indicate shared targets of EQF and NAFLD, while dark orange nodes represent targets unique to EQF. (**B**) PPI network of common EQF–NAFLD targets. Node color intensity and diameter reflect the degree of connectivity in the network. Darker and larger nodes correspond to more critical targets. Edges indicate protein–protein interactions. (**C**) GO enrichment bubble plot of DEGs. (**D**) KEGG enrichment bubble plot of DEGs. (**E**) Relative mRNA expression levels of BCL-2, Caspase3, ESR1, STAT3, and TNF were measured by RT-qPCR. (**F**) Representative Western blot bands and quantification of Caspase3, BCl-2, c-Jun, p-STAT3, and STAT3 proteins. Data are presented as mean ± SD. ^###^ *p* < 0.001 vs. control group; * *p* < 0.05, ** *p* < 0.01, *** *p* < 0.001 vs. model group. Model: NAFLD group; EQF-L group: 11.18 g·kg^−1^·d^−1^; EQF-H group: 22.36 g·kg^−1^·d^−1^; Feno group: 0.026 g·kg^−1^·d^−1^.

Network pharmacology analysis identified 73 potential therapeutic targets of EQF against NAFLD, obtained by intersecting 234 compound-related targets (SwissTargetPrediction) with 2833 NAFLD-associated genes (GeneCards, OMIM, DisGeNET). The compound–target network (Figure 2A) revealed six key active components, including aloe-emodin, rhein, and daidzein. From the PPI network of common targets (Figure 2B), seven core targets were screened: BCL2, CASP3, ESR1, JUN, MMP9, STAT3, and TNF. GO and KEGG enrichment analyses indicated that these targets were significantly involved in biological processes such as aging and response to lipopolysaccharide, and were enriched in pathways including AGE-RAGE, IL-17, lipid and atherosclerosis, and TNF signaling (Figure 2C,D).

To further elucidate the regulatory effects of EQF on key targets in NAFLD, RT-qPCR and Western blot were used to assess the mRNA and protein expression levels of BCL2, CASP3, ESR1, JUN, MMP9, STAT3, and TNF.

Western blot results demonstrated that EQF treatment significantly up-regulated BCL-2 (*p* < 0.05) while concurrently down-regulating the expression of Caspase3 (*p* < 0.01) and the inflammatory mediator c-Jun (*p* < 0.05), and EQF administration effectively suppressed STAT3 activation, as evidenced by the reduced p-STAT3/STAT3 ratio (*p* < 0.05). The mRNA expression patterns consistently revealed that EQF treatment enhanced the mRNA expression of the protective genes BCL-2 and ESR1 (*p* < 0.05), while simultaneously suppressing the transcript levels of Caspase3 and STAT3 and TNF followed a dose-responsive pattern (*p* < 0.05). The positive control drug Feno showed a similar effect on the above indexes (*p* < 0.05), and there was no statistical difference between the p-STAT3/STAT3 ratio and the mRNA expression levels of ESR1 (Figure 2E,F).

### 3.3. Molecular Docking Validation of EQF Components and Targets

**Figure 3:**
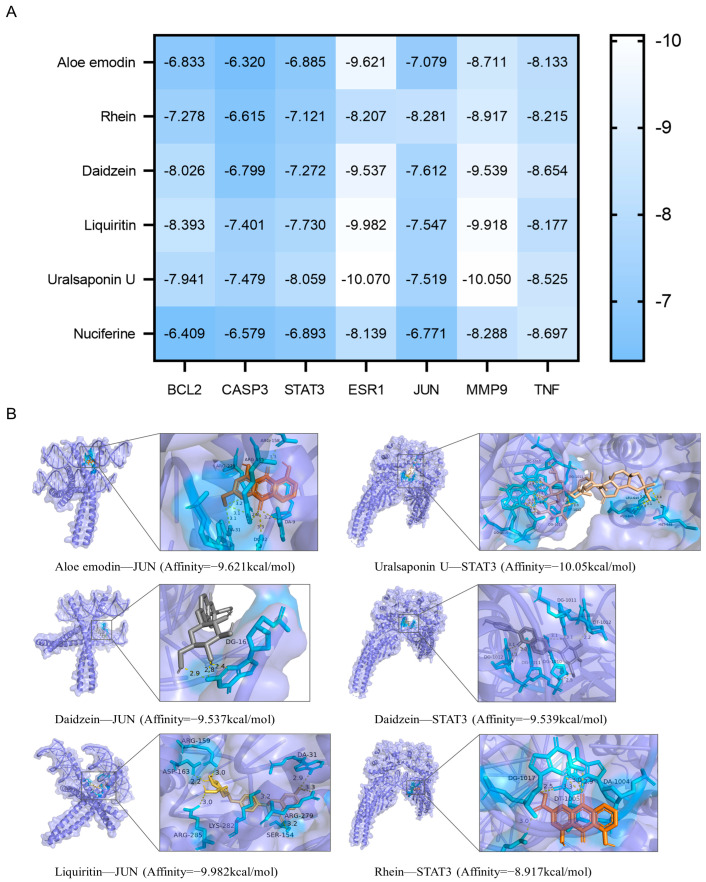
Molecular docking results of active components and key targets of EQF against NAFLD. (**A**) The binding energy scores (kcal/mol). (**B**) Molecular docking analysis for the major constituents of EQF binding to JUN and STAT3 proteins. The main components of EQF: aloe-emodin, rhein, daidzein, liquiritin, uralsaponin U, and nuciferine. The receptor proteins are coloured purple, key amino-acid residues in the binding pocket are shown as cyan sticks, and the docked ligands are shown as orange sticks; hydrogen bonds are indicated by dashed lines.

Molecular docking predicts the binding modes and affinity between receptors and ligands using structural information. A lower binding energy generally corresponds to a more stable interaction. In this study, molecular docking analysis was performed to evaluate the binding capacity of core EQF components to key targets. The docking models and binding energy scores are summarized in Figure 3A. Specifically, the binding energies between all key targets and core components were lower than −6 kcal/mol, indicating favorable binding stability. Based on molecular docking analysis, JUN and STAT3 emerged as the primary targets due to their superior binding energies with all six core components of EQF (Figure 3B), indicating their potential role as key targets in mediating the drug’s effects.

### 3.4. The Therapeutic Effect of EQF on NAFLD Model

**Figure 4:**
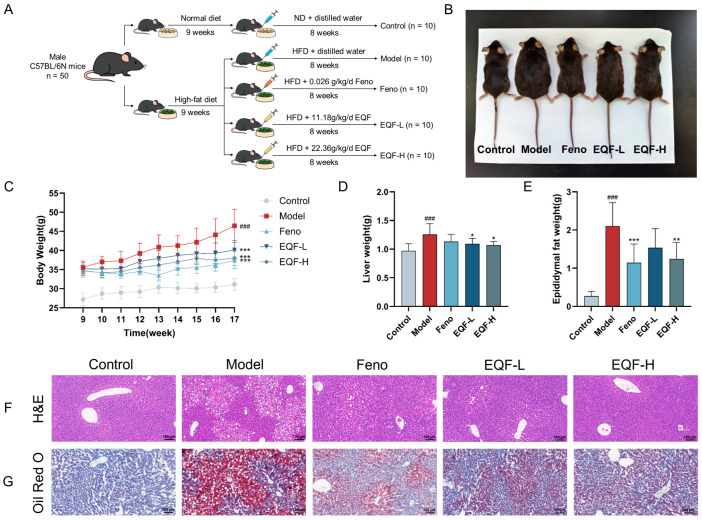
Erhuang Quzhi Formula alleviates hepatic lipid accumulation in NAFLD mice. (**A**) Schematic diagram of the animal experimental design. (**B**) Representative photographs showing body morphology of mice in each group (*n* = 10). (**C**) Statistical analysis of body weight changes in each group (*n* = 10). (**D**) Statistical analysis of liver weight in each group (*n* = 10). (**E**) Statistical analysis of epididymal fat weight in each group (*n* = 10). (**F**) Representative H&E-staining images of liver tissues (*n* = 3, scale bar: 100 μm). (**G**) Representative Oil Red O-staining images of liver tissues (*n* = 3, scale bar: 100 μm). Data are presented as mean ± SD. ^###^ *p* < 0.001 vs. control group; * *p* < 0.05, ** *p* < 0.01, *** *p* < 0.001 vs. model group. ND: normal diet. HFD: high-fat diet.

To evaluate the therapeutic potential of EQF against NAFLD, a mouse model was established by feeding C57BL/6J mice a HFD for 10 weeks. Animals were divided into treatment groups receiving either EQF at different doses or Feno as a positive control, with the experimental design outlined in Figure 4A. In the model group, body weight and organ weight (liver and epididymal fat) significantly increased compared to the control group (*p* < 0.001). EQF treatment suppressed weight gain in a dose-dependent manner, with the EQF-H group showing the most substantial effect (*p* < 0.001). Feno exhibited the strongest overall weight control (*p* < 0.001). Both EQF and Feno significantly reversed HFD-induced increases in liver and fat mass, with EQF-H particularly effective in reducing liver weight (*p* < 0.05) and Feno the most effective in reducing epididymal fat (*p* < 0.001) (Figure 4B–E).

Histopathological analysis further confirmed the therapeutic potential of EQF. H&E staining demonstrated that EQF treatment significantly ameliorated HFD-induced hepatic structural disorganization, reducing hepatocyte ballooning and inflammatory infiltration while restoring hepatic cord architecture, with the most prominent improvement observed in the EQF-H group. Similarly, Oil Red O staining revealed a substantial reduction in both the area and intensity of lipid droplets in EQF-treated groups, particularly in EQF-H, comparable to the improvement seen in the Feno group (Figure 4F,G). In summary, EQF effectively attenuated HFD-induced hepatic steatosis, inflammation, and structural damage, demonstrating dose-dependent hepatoprotective effects against NAFLD.

### 3.5. Effects of EQF on Metabolic Parameters and Liver Function in NAFLD Mice

**Figure 5:**
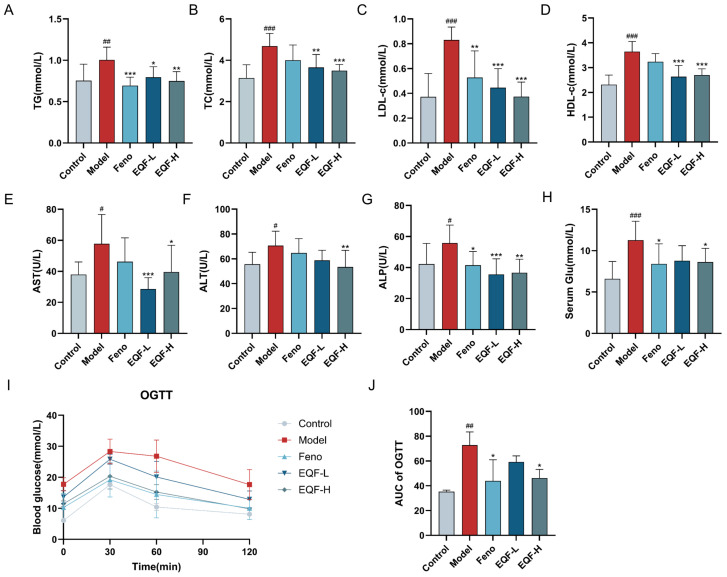
EQF improves serum biochemical parameters in HFD-induced NAFLD mice. (**A**) TG. (**B**) TC. (**C**) LDL-C. (**D**) HDL-C. (**E**) AST. (**F**) ALT. (**G**) ALP. (**H**) Glu. (**I**) OGTT curve. (**J**) AUC. (*n* = 10) Data are presented as mean ± SD. ^#^ *p* < 0.05, ^##^ *p* < 0.01, ^###^ *p* < 0.001 vs. control group; * *p* < 0.05, ** *p* < 0.01, *** *p* < 0.001.vs. model group.

To evaluate the regulatory effect of EQF on lipid metabolism, serum lipid parameters—including TG, TC, LDL-C, and HDL-C—were measured (Figure 5A–D). Compared with the control group, HFD-fed mice exhibited significant increases in all four lipid parameters. EQF treatment dose-dependently reduced TG, TC, and LDL-C levels (*p* < 0.05). Although HDL-C was also significantly decreased by EQF, no clear dose–response relationship was observed (*p* < 0.05). The Feno group significantly lowered TG and LDL-C (*p* < 0.01), but did not significantly affect TC or HDL-C.

To assess the hepatoprotective effect of EQF, serum liver enzyme activities (AST, ALT, ALP) were measured in NAFLD mice. The model group exhibited significantly elevated levels of all three enzymes compared to the control group (*p* < 0.05), confirming the successful induction of liver injury (Figure 5E–G). EQF intervention effectively reversed these abnormalities in a marker-specific manner: both EQF-L and EQF-H significantly reduced AST and ALP levels (*p* < 0.05), while only the EQF-H group produced a statistically significant ALT reduction (*p* < 0.05). In contrast, the Feno group only significantly decreased ALP (*p* < 0.05), without showing notable effects on AST or ALT.

Fasting blood glucose levels were significantly elevated in the model group compared to the control group, confirming the successful induction of hyperglycemia by a HFD (*p* < 0.01). EQF-H group intervention significantly reduced blood glucose to levels comparable with the Feno group (*p* < 0.05) (Figure 5H). In the oral glucose tolerance test, the model group exhibited significantly higher blood glucose at all time points after the glucose challenge, and failed to return to baseline by 120 min (*p* < 0.01), indicating severely impaired glucose tolerance. All EQF dosage groups effectively lowered the blood glucose peak and enhanced clearance, with the EQF-H group showing the most substantial improvement, similar to the Feno group (*p* < 0.05) (Figure 5I,J).

These results demonstrate that EQF can effectively alleviate HFD-induced liver function abnormalities and dose-dependently improve glucose tolerance impairment and hyperglycemia in mice, showing good blood glucose regulatory activity.

### 3.6. Regulatory Effects of EQF on Hepatic Lipids

**Figure 6:**
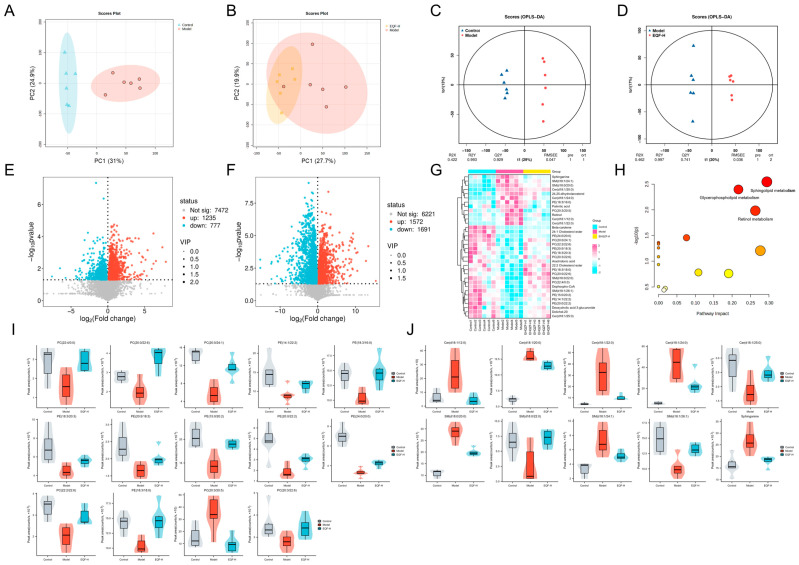
Hepatic lipidomic profiling. PCA score plots (**A**) between control and model groups; (**B**) between model and EQF-H groups. OPLS-DA score plot (**C**) between control and model groups; (**D**) between model and EQF-H groups. Volcano plot of differential lipid molecules (**E**) between control and model groups; (**F**) between model and EQF-H groups. (**G**) Differential lipid molecular cluster heat map analysis of control, model and EQF-H groups. (**H**) KEGG pathway enrichment analysis. In the bubble plot, each circle represents a KEGG pathway; the colour (yellow to red) reflects the pathway enrichment significance, and the circle size is proportional to the pathway impact. (**I**) Peak area of differential glycerophospholipids in lipidomics. (**J**) Peak area of differential sphingolipids in lipidomics (*n* = 6).

To further investigate the lipid metabolism regulatory mechanisms and related biomarkers underlying the amelioration of NAFLD by EQF, a lipidomics analysis was performed on liver tissues from control-, model-, and EQF-H-group mice. Principal component analysis (PCA) showed that the first two principal components (PC1 and PC2) accounted for 55.9% of the total variance in the model versus control comparison and 47.6% in the EQF-H versus model comparison. Although these values were below the conventional threshold of 70%, which was often observed in lipidomics studies due to biological complexity and individual variability, a clear separation trend along the PC1 axis was still observed among groups. This indicates that the model effectively captured key variations induced by the experimental intervention. Specifically, the PCA score plot revealed distinct separation between the model and control groups along PC1, suggesting that the high-fat diet induced hepatic lipid metabolic disorders. In the EQF-H group, samples shifted toward the control group cluster along the PC1 axis, with a more compact distribution and reduced dispersion (Figure 6A,B). These results indicate that EQF intervention reduced within-group variability and restored the abnormal lipid profile to a normal state.

Orthogonal projections to latent structures–discriminant analysis (OPLS-DA) was further employed to validate the intergroup differences in lipid profiles. The comparison model between the control and model groups demonstrated high model stability and predictive ability (R^2^Y = 0.993, Q^2^Y = 0.929). OPLS-DA score plots revealed clear separation between these groups, confirming that HFD successfully induced global perturbations in hepatic lipid metabolism. Similarly, the model comparing the EQF-H and model groups also showed good stability and predictive performance (R^2^Y = 0.997, Q^2^Y = 0.741). The distinct separation trend indicated that EQF intervention effectively restored the HFD-induced metabolic disorder (Figure 6C,D).

Based on the criteria of fold change (FC) ≥ 1, VIP ≥ 1, and *p* < 0.05, differential lipid molecules were screened from inter-group comparisons. Volcano plots (Figure 6E,F) revealed that compared to the control group, 3263 lipids were significantly altered in the model group (1572 up-regulated, 1691 down-regulated). In the EQF-H group, 2012 lipids showed significant changes compared to the model group (1235 up-regulated, 777 down-regulated). Notably, 34 lipids that were differentially expressed in the model group were notably reversed after EQF-H intervention, returning to levels comparable to the control group (Figure 6G).

Metabolic pathway analysis of the 34 key differential lipids using MetaboAnalyst 6.0 revealed sphingolipid metabolism and glycerophospholipid metabolism as the most significantly enriched pathways, both closely associated with NAFLD (Figure 6H). Lipidomic profiling demonstrated extensive disruptions in these pathways in model group mice: glycerophospholipid metabolism showed 1 significantly increased and 13 decreased lipids (Figure 6I), while sphingolipid metabolism exhibited 7 elevated and 3 reduced lipids. EQF-H treatment significantly reversed all these dysregulated lipid levels (Figure 6J), indicating EQF’s effectiveness in restoring lipid metabolic homeostasis.

### 3.7. Regulatory Effects of EQF on Hepatic Metabolism

**Figure 7:**
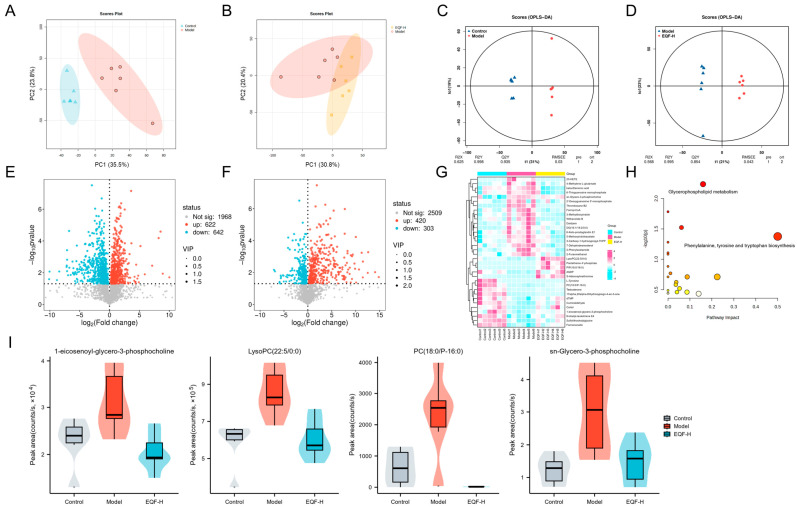
Hepatic metabolomic profiling. PCA score plots (**A**) between control and model groups; (**B**) between model and EQF-H groups. OPLS-DA score plot (**C**) between control and model groups; (**D**) between model and EQF-H groups. Volcano plot of differential lipid molecules (**E**) between control and model groups; (**F**) between model and EQF-H groups. (**G**) Differential lipid molecular cluster heat map analysis of control, model and EQF-H groups. (**H**) KEGG pathway enrichment analysis. In the bubble plot, each circle represents a KEGG pathway; the colour (yellow to red) reflects the pathway enrichment significance, and the circle size is proportional to the pathway impact. (**I**) Peak area of differential glycerophospholipid metabolites in metabolomics (*n* = 6).

This study utilized metabolomics approaches to systematically evaluate the regulatory effects of EQF on hepatic metabolism in NAFLD mice. Principal component analysis (PCA) of hepatic metabolomic data showed that PC1 and PC2 explained 59.3% of the total variance in the model versus control comparison and 51.2% in the EQF-H versus model comparison. Although these values were below the conventional threshold, the PCA score plot displayed clear separation between the model and control groups. The cluster centers were distinctly separated, and confidence ellipses showed no overlap. These results indicate that the HFD induced a global metabolic disturbance in the liver (Figure 7A,B). In contrast, the EQF-H group separated from the model group along the principal component direction. Its sample points moved closer to the control group and displayed a more compact distribution, suggesting that EQF-H treatment alleviated the metabolic disturbances induced by HFD.

OPLS-DA further confirmed substantial intergroup metabolic differences. The model and control groups showed clear separation (R^2^Y = 0.998, Q^2^Y = 0.935), indicating significant metabolic alterations in NAFLD mice (Figure 7C). Similarly, the EQF-H group demonstrated significant separation from the model group (R^2^Y = 0.995, Q^2^Y = 0.854), reflecting EQF’s efficacy in ameliorating metabolic abnormalities (Figure 7D).

FC > 1, VIP > 1, *p* < 0.05 identified 1264 altered metabolites in the model group compared to the control group (622 up- and 642 down-regulated). The EQF-H group showed 723 altered metabolites compared to the model group (420 up- and 303 down-regulated). Notably, 34 metabolites that were abnormally expressed in the model group were significantly reversed by EQF-H intervention, returning to near-normal levels (Figure 7E–G).

Pathway enrichment analysis of these 34 key metabolites identified glycerophospholipid metabolism as the most significantly affected pathway, which is closely associated with NAFLD pathogenesis (Figure 7H). Within this pathway, four representative glycerophospholipid metabolites were significantly elevated in the model group but restored to near-normal levels following EQF-H intervention (Figure 7I), suggesting that modulation of glycerophospholipid metabolism may represent a crucial mechanism underlying EQF’s therapeutic effects.

## 4. Discussion

NAFLD is a complex metabolic-stress-induced liver disorder characterized by intricate pathological mechanisms. It is not only closely associated with hepatic lipid metabolism dysregulation, insulin resistance, oxidative stress, and inflammatory responses [26], but also represents a hepatic manifestation of systemic metabolic syndrome. Currently, no specific therapeutic agents have been approved for NAFLD treatment in clinical practice [30,31]. The existing strategies primarily focus on lifestyle interventions and the management of comorbidities, which offer limited efficacy and are often accompanied by certain side effects [32]. Consequently, there is an urgent need to develop safe and effective pharmacological treatments. In this context, TCM, with its holistic perspective and multi-target regulatory capabilities, has demonstrated substantial therapeutic potential [33]. EQF, a classic TCM formula, has attracted considerable attention due to its potential hepatoprotective and metabolic-modulating effects. However, the specific pharmacodynamic material basis and molecular mechanisms underlying its efficacy against NAFLD remain unclear, which has limited its clinical application [34]. This study employed an integrated approach combining serum pharmacochemistry, network pharmacology, molecular docking, in vivo experimental validation, and multi-omics analysis to systematically elucidate the multi-component, multi-target, and multi-pathway mechanisms of EQF in treating NAFLD.

The chemical profile of the EQF decoction and the analysis of drug-containing serum together clarify its in vivo exposure and potential bioactive constituents. In this study, 83 compounds were identified in the EQF decoction. However, only 31 prototype components were detected in the serum after oral administration. This difference underscores a key principle in herbal pharmacology: the in vitro composition does not fully represent the in vivo situation, as bioavailability plays a critical role [35,36]. The 31 absorbed prototypes, mainly flavonoids and quinones, represent potential active constituents of EQF, although contributions from other minor metabolites and uncharacterized components cannot be excluded. Their presence in serum confirms their absorption and potential to reach target tissues.

Furthermore, 75 metabolites were identified, most formed through Phase II conjugation reactions such as glucuronidation and sulfation. These metabolites, though not present in the original decoction, may exhibit modified bioactivities and contribute to the therapeutic effects of EQF. This integrated approach helps link the complex chemical profile of EQF to its pharmacological behavior. The combination of absorbed prototypes and their metabolites forms the effective material basis of EQF against NAFLD. Future studies should focus on these serum components, particularly the synergistic actions and roles of key metabolites, to further clarify the mechanism of EQF in NAFLD treatment.

Integrated network pharmacology analysis further screened six key active compounds (aloe-emodin, rhein, daidzein, nuciferine, liquiritin, and uralsaponin U) from the aforementioned components, which collectively constitute a “multi-component” synergistic ensemble against NAFLD. Subsequent PPI network and topological analyses prioritized 7 putative core targets from 73 potential targets: BCL2, CASP3, ESR1, JUN, MMP9, STAT3, and TNF. These targets are not isolated; they are functionally interlinked, forming a critical network closely associated with apoptosis, inflammatory response, and hormone signal transduction. These predictions were preliminarily supported at the expression level by Western blot and RT-qPCR experiments. Molecular docking provided structural evidence at the atomic level. The results indicated that all six core components exhibited favorable predicted binding (binding energies < −6 kcal/mol) with the seven key targets. Notably, the key components showed relatively stronger predicted binding energies with JUN and STAT3, suggesting relatively stable interactions. This strongly suggests that JUN and STAT3 may serve as important potential molecular hubs where the multiple components of EQF are predicted to converge and exert their regulatory effects.

The core targets identified in this study are not randomly distributed but are highly concentrated in apoptosis and inflammatory signaling pathways. For instance, TNF, as a key initiator of inflammatory responses, can activate JUN and STAT3 signaling [37], which in turn regulates the balance between downstream CASP3 (pro-apoptotic) and BCL2 (anti-apoptotic), ultimately determining hepatocyte fate [38]. Molecular docking results showed that multiple components of EQF are predicted to bind with relatively high affinity to key targets within the signaling axis. These findings provide structural support consistent with the observed anti-inflammatory and anti-apoptotic effects of EQF. Traditional Chinese medicine formulas often exert therapeutic effects through multi-component synergy. The “6-component, 7-target” network identified in this study reflects this synergistic mechanism. For example, aloe-emodin and rhein potentially inhibit inflammatory targets (such as TNF) [39,40], while daidzein and liquiritin may cooperatively regulate apoptotic signals (e.g., BCL2/CASP3) [41]. This multi-target, systematic regulatory mode enables EQF to implement multi-layered and comprehensive intervention against the complex pathological network of NAFLD. The progression of NAFLD is characterized by exacerbated hepatocyte apoptosis/necrosis and chronic low-grade inflammation. The core targets screened in this study are considered to function as important regulators of these two central pathological processes [42,43]. Through its multi-component system, EQF acts on this regulatory network, thereby potentially curbing disease progression at its root rather than merely alleviating symptoms. This study integrates the “material identification” from serum pharmacochemistry with the “mechanistic prediction” from network pharmacology through the “structural hypotheses” provided by molecular docking, forming an integrated chain of evidence [19,44]. However, we acknowledge that functional inhibition or genetic manipulation of these targets was not performed in the present study, and future work will be required to confirm their causal roles in mediating the protective effects of EQF. This approach helps to elucidate potential mechanism of action of EQF and provides a mechanistic framework that may facilitate the development of TCM formulations into modern drugs with well-defined targets.

In vivo experiments demonstrated the therapeutic potential of EQF against NAFLD. Compared with the NAFLD group, EQF administration significantly suppressed HFD-induced body weight gain in a dose-dependent manner. EQF also reduced liver weight and epididymal fat weight, indicating the alleviation of obesity and hepatomegaly, which are core features of NAFLD. Histopathological analysis showed that EQF markedly ameliorated hepatic lesions, including lipid vacuolation, ballooning degeneration, and inflammatory cell infiltration. Hepatocyte morphology was also restored.

Moreover, EQF improved systemic metabolic parameters. It significantly decreased serum levels of TG, TC, and LDL-C, and reduced the activities of AST, ALT, and ALP, suggesting repair of hepatocellular injury. NAFLD is often characterized by concurrent glucose and lipid metabolism disorders. The results indicated that EQF regulates glucose homeostasis, which may indirectly reduce hepatic lipid accumulation. These findings reflect the multi-target intervention characteristics of traditional Chinese medicine.

The in vivo experimental results of this study not only confirm the therapeutic efficacy of EQF, but also comparison with the positive control drug Feno, suggest the potential value of multi-target intervention by TCM compounds. While Feno demonstrated stronger effects on certain individual indicators such as body weight reduction, TG lowering, and oral glucose tolerance improvement, EQF, particularly at the high dose, showed broadly comparable improvements in several metabolic parameters, with particularly significant advantages in lowering liver injury markers (AST, ALT) [45]. This difference reflects a fundamental distinction between the two therapeutic strategies: Feno aims for “precision repair” of specific pathways, whereas EQF tends to perform “ecological regulation” of the body’s metabolic environment, thereby remodeling the dynamic balance among lipid metabolism, inflammation, and blood glucose through multi-target synergy. This holistic therapeutic effect is underpinned by a profound molecular basis. The core targets related to apoptosis and inflammation (e.g., TNF, CASP3, JUN, STAT3) [46], previously predicted by network pharmacology, were functionally corroborated by the observed alleviation of hepatic inflammatory infiltration [47] and repair of hepatocyte damage in this study. This confirms that the active components in the serum collectively translate into tangible hepatoprotective effects by acting on this target network. Furthermore, this discovery reveals the considerable clinical potential of multi-target compounds like EQF in addressing complex metabolic syndromes such as NAFLD. Their “multi-pronged” approach aligns well with the clinical management needs of patients who often present with multiple metabolic disorders [48]. Based on these findings, the combination of EQF and fenofibrate may offer complementary therapeutic benefits. Further investigation is warranted to evaluate the potential synergistic effects and safety profile of this combined regimen.

To systematically elucidate the deep pharmacological mechanisms of EQF in intervening in NAFLD, this study adopted an integrated lipidomic and metabolomics analysis strategy to investigate its metabolic regulatory effects at the biological level. The results demonstrated that EQF comprehensively and systematically reversed the disturbances in hepatic lipid profiles and serum metabolic profiles induced by an HFD. Specifically, 34 key lipid molecules and 34 endogenous metabolites were identified, all of which showed significant aberrant expression in the model group but were markedly reversed to near-normal levels following EQF intervention. These molecules collectively form a key metabolic regulatory network through which EQF exerts its effects. Further pathway enrichment analysis revealed that sphingolipid metabolism and glycerophospholipid metabolism were the two most significantly regulated core metabolic pathways by EQF. Sphingolipids (such as ceramides) [49] are not only essential components of cell membrane structure, but are also well-established lipotoxic molecules and inflammatory signaling mediators [50,51]. In NAFLD, especially NASH, the accumulation of sphingolipids such as ceramides is closely related to liver inflammation and metabolic disorders [52]. Glycerophospholipids, as the primary structural basis of biological membranes [53], when metabolically imbalanced, can compromise hepatocyte membrane integrity and disrupt the assembly and secretion of VLDL, thereby promoting intrahepatic lipid deposition [54]. Alterations in phosphatidylcholine and phosphatidylethanolamine have been linked to difficulties in VLDL secretion, which can result in fat buildup in the liver, a condition known as steatosis, and may lead to the development of NAFLD [55,56].

By coordinately regulating these two pathologically interconnected lipid metabolic axes, EQF alleviates hepatic lipotoxic stress and metabolic burden at the source, which likely constitutes the core biochemical mechanism behind its comprehensive hepatoprotective effects. Importantly, untargeted metabolomics analysis independently identified glycerophospholipid metabolism as one of the most affected pathways, highly consistent with the lipidomics findings, providing strong cross-methodological validation [57]. This convergent evidence collectively indicates that EQF possesses a powerful capacity to remodel hepatic lipid metabolic homeostasis, thereby furnishing solid system-level scientific basis for understanding its holistic therapeutic characteristics of being “multi-component, multi-target, and multi-pathway.” However, all experiments were performed in a mouse model of NAFLD induced by a high-fat diet. Due to species differences in pharmacokinetics, hepatic metabolism, and target regulation, these preclinical results may not be directly applicable to humans. Further clinical studies are needed to verify the translational potential of EQF.

## 5. Conclusions

In conclusion, this study establishes an integrated framework from chemical composition to serum-absorbed components, predicted targets, in vivo efficacy, and key pathways, clarifying the multidimensional mechanisms of EQF against NAFLD in mice. The results indicate that EQF, likely through synergistic effects of multiple serum-absorbed bioactive compounds, regulates key targets such as JUN and STAT3. These actions correlate with improved hepatic steatosis, reduced inflammation and liver injury, and restored systemic glucose and lipid metabolic homeostasis. Potential contributions from undetected or minor metabolites remain possible. The core mechanism involves systematic reprogramming of dysregulated hepatic metabolism, particularly in sphingolipid and glycerophospholipid pathways. These findings provide a systematic mechanistic basis for the effects of EQF in a NAFLD mouse and a reference model for studying multi-component, multi-target traditional Chinese medicine formulations.

## Figures and Tables

**Table 1:** Identification of chemical components in EQF decoction and prototype components in rat serum after oral administration of EQF.

No.	Component Name	Detected in Serum	Molecular Formula	Adducts	Expected Mass *m*/*z*	Observed	Error (ppm)	t_R_ (min)	Database Match
1	Betaine		C_5_H_11_NO_2_	[M + H]^+^	118.0863	118.0862	0.8	1.03	√
2	Citric acid		C_6_H_8_O_7_	[M − H]^−^	191.0197	191.0198	−0.5	2.10	√
3	Adenosine		C_10_H_13_N_5_O_4_	[M + H]^+^	268.1040	268.1040	0.0	3.74	√
4	Guanosine		C_10_H_13_N_5_O_5_	[M + H]^+^	284.0989	284.0991	−0.7	4.61	√
5	Methyl (1α,3R,4α,5R)-1,3,4,5-tetrahydroxycyclohexanecarboxylate		C_8_H_14_O_6_	[M − H]^−^	205.0718	205.0721	−1.5	7.97	
6	Atractyloside A		C_21_H_36_O_10_	[M + FA − H]^−^	493.2300	493.2299	0.2	10.34	√
7	Coclaurine		C_17_H_19_NO_3_	[M + H]^+^	286.1439	286.1440	−0.3	10.90	
8	Puerarin-4′-O-glucoside	√	C_27_H_30_O_14_	[M − H]^−^	577.1563	577.1562	0.2	10.94	
9	Hirudonucleodisulfide B		C_11_H_10_N_4_O_4_S_2_	[M − H]^−^	325.0077	325.0060	5.2	11.02	
10	*N*-Methylcoclaurine		C_18_H_21_NO_3_	[M + H]^+^	300.1594	300.1598	−1.3	11.14	
11	Catechin		C_15_H_14_O_6_	[M − H]^−^	289.0718	289.0716	0.7	11.39	√
12	Aloe-emodin-8-glucopyranoside	√	C_21_H_20_O_10_	[M − H]^−^	431.0984	431.0982	0.5	12.21	√
13	Daidzein-4′,7-diglucoside	√	C_27_H_30_O_14_	[M + FA − H]^−^	623.1618	623.1623	−0.8	12.32	
14	3′-Hydroxypuerarin 7-O-sulfate	√	C_21_H_20_O_13_S	[M − H]^−^	511.0552	511.0554	−0.4	13.02	
15	Roseoside		C_19_H_30_O_8_	[M + FA − H]^−^	431.1923	431.1921	0.5	13.38	
16	Armepavine		C_19_H_23_NO_3_	[M + H]^+^	314.1751	314.1754	−1.0	13.52	√
17	6″-O-Apiofuranosyl-3′-hydroxypuerarin		C_26_H_28_O_14_	[M − H]^−^	563.1406	563.1414	−1.4	13.62	
18	*N*-Norarmepavine	√	C_18_H_21_NO_3_	[M + H]^+^	300.1594	300.1598	−1.3	13.97	
19	Hirudonucleodisulfide A		C_10_H_6_N_4_O_4_S_2_	[M − H]^−^	308.9758	308.9754	1.3	14.04	
20	Epicatechin		C_15_H_14_O_6_	[M − H]^−^	289.0718	289.0715	1.0	14.12	√
21	Puerarin	√	C_21_H_20_O_9_	[M − H]^−^	415.1035	415.1030	1.2	14.25	√
22	3′-Methoxypuerarin	√	C_22_H_22_O_10_	[M − H]^−^	445.1140	445.1136	0.9	15.01	√
23	6″-O-Xylosylpuerarin	√	C_26_H_28_O_13_	[M − H]^−^	547.1457	547.1459	−0.4	15.14	
24	Mirificin	√	C_26_H_28_O_13_	[M − H]^−^	547.1457	547.1455	0.4	15.54	
25	Daidzin	√	C_21_H_20_O_9_	[M + FA − H]^−^	461.1089	461.1092	−0.7	16.69	√
26	3′-Methoxydaidzin		C_22_H_22_O_10_	[M + FA − H]^−^	491.1195	491.1199	−0.8	17.59	
27	6″-O-Apiofuranosyl-Genistein 8-C-glucoside	√	C_26_H_28_O_14_	[M − H]^−^	563.1406	563.1415	−1.6	18.26	
28	Polydatin	√	C_20_H_22_O_8_	[M − H]^−^	389.1242	389.1240	0.5	18.60	
29	Liquiritin	√	C_21_H_22_O_9_	[M − H]^−^	417.1191	417.1187	1.0	18.68	√
30	Liquiritin apioside	√	C_26_H_30_O_13_	[M − H]^−^	549.1614	549.1610	0.7	19.04	
31	Rutin		C_27_H_30_O_16_	[M − H]^−^	609.1461	609.1464	−0.5	19.57	√
32	Isoquercitrin	√	C_21_H_20_O_12_	[M − H]^−^	463.0882	463.0882	0.0	20.01	√
33	Nuciferine	√	C_19_H_21_NO_2_	[M + H]^+^	296.1645	296.1650	−1.7	20.37	√
34	Quercetin-3-O-β-D-glucuronide	√	C_21_H_18_O_13_	[M − H]^−^	477.0675	477.0669	1.3	20.50	√
35	Isochlorogenic acid B		C_25_H_24_O_12_	[M − H]^−^	515.1195	515.1202	−1.4	21.46	√
36	6″-O-Malonyldaidzin		C_24_H_22_O_12_	[M + H]^+^	503.1184	503.1195	−2.2	21.46	
37	Salvianolic acid J		C_27_H_22_O_12_	[M − H]^−^	537.1039	537.1040	−0.2	21.50	
38	Salvianolic acid D		C_20_H_18_O_10_	[M − H]^−^	417.0827	417.0824	0.7	21.62	√
39	Isochlorogenic acid A		C_25_H_24_O_12_	[M − H]^−^	515.1195	515.1199	−0.8	21.71	√
40	Rosmarinic acid		C_18_H_16_O_8_	[M − H]^−^	359.0772	359.0764	2.2	22.85	√
41	Isochlorogenic acid C		C_25_H_24_O_12_	[M − H]^−^	515.1195	515.1185	1.9	23.02	√
42	6″-O-Acetyldaidzin		C_23_H_22_O_10_	[M + H]^+^	459.1286	459.1293	−1.5	23.11	
43	Salvianolic acid E		C_36_H_30_O_16_	[M − H]^−^	717.1461	717.1460	0.1	23.59	√
44	Formononetin glucoside	√	C_22_H_22_O_9_	[M + H]^+^	431.1337	431.1342	−1.2	23.66	√
45	Lithospermic acid		C_27_H_22_O_12_	[M − H]^−^	537.1039	537.1042	−0.6	23.91	√
46	Daidzein	√	C_15_H_10_O_4_	[M − H]^−^	253.0506	253.0504	0.8	24.59	√
47	Salvianolic acid B		C_36_H_30_O_16_	[M − H]^−^	717.1461	717.1456	0.7	25.52	√
48	Morin		C_15_H_10_O_7_	[M − H]^−^	301.0354	301.0350	1.3	26.48	√
49	Salvianolic acid Y		C_36_H_30_O_16_	[M − H]^−^	717.1461	717.1460	0.1	26.56	√
50	Emodin 8-O-glucoside	√	C_21_H_20_O_10_	[M − H]^−^	431.0984	431.0986	−0.5	27.15	√
51	Formononetin-7-*O*-β-D-glucoside-6″-*O*-malonate		C_25_H_24_O_12_	[M + H]^+^	517.1341	517.1353	−2.3	27.25	
52	Bisdemethoxycurcumin		C_19_H_16_O_4_	[M − H]^−^	307.0976	307.0974	0.7	29.90	√
53	Emodin-8-O-(6-malonyl)-glucoside		C_24_H_22_O_13_	[M − H]^−^	517.0988	517.0991	−0.6	30.17	
54	22-Hydroxy-licorice saponin G2		C_42_H_62_O_18_	[M + H]^+^	855.4009	855.4044	−4.1	30.31	
55	Isocurcumin		C_21_H_20_O_6_	[M + H]^+^	369.1333	369.1344	−3.0	30.94	
56	Licorice saponin A3	√	C_48_H_72_O_21_	[M − H]^−^	983.4493	983.4500	−0.7	31.05	
57	Gypenoside XLIII		C_54_H_92_O_23_	[M + FA − 2H]2^−^	599.2997	599.3014	−2.8	31.25	
58	Licoricesaponin G2	√	C_42_H_62_O_17_	[M − H]^−^	837.3914	837.3916	−0.2	31.82	
59	16-Oxoalisol A	√	C_30_H_48_O_6_	[M + FA − H]^−^	549.3433	549.3440	−1.3	32.53	√
60	Gypenoside XLVI		C_48_H_82_O_19_	[M + FA − H]^−^	1007.5432	1007.5449	−1.7	32.82	√
61	6-Methylrhein	√	C_16_H_10_O_6_	[M − H]^−^	297.0405	297.0403	0.7	32.87	
62	Gypenoside XIX		C_54_H_92_O_23_	[M − H]^−^	1107.5957	1107.5982	−2.3	33.03	
63	Uralsaponin U	√	C_42_H_62_O_17_	[M − H]^−^	837.3914	837.3923	−1.1	33.40	
64	Rhein	√	C_15_H_8_O_6_	[M − H]^−^	283.0248	283.0247	0.4	33.58	√
65	Ginsenoside Re		C_48_H_82_O_18_	[M + FA − H]^−^	991.5483	991.5525	−4.2	33.91	√
66	Macedonoside A		C_42_H_62_O_17_	[M − H]^−^	837.3914	837.3929	−1.8	34.26	
67	Glycyrrhizic acid	√	C_42_H_62_O_16_	[M − H]^−^	821.3965	821.3973	−1.0	34.39	√
68	Gypenoside VII		C_54_H_92_O_21_	[M + FA − H]^−^	1121.6113	1121.6142	−2.6	34.68	
69	Atractylenolide I		C_15_H_18_O_2_	[M + H]^+^	231.1380	231.1386	−2.6	34.97	√
70	Uralsaponin B		C_42_H_62_O_16_	[M − H]^−^	821.3965	821.3970	−0.6	35.34	
71	Ginsenoside Rg2		C_42_H_72_O_13_	[M + FA − H]^−^	829.4955	829.4957	−0.2	36.43	√
72	Demethoxycyclocurcumin		C_20_H_18_O_5_	[M − H]^−^	337.1081	337.1081	0.0	36.71	√
73	Curcumin		C_21_H_20_O_6_	[M + H]^+^	369.1333	369.1344	−3.0	37.12	√
74	Aloe-emodin	√	C_15_H_10_O_5_	[M − H]^−^	269.0455	269.0456	−0.4	37.42	√
75	Alisol C,23-acetate		C_32_H_48_O_6_	[M + FA − H]^−^	573.3433	573.3440	−1.2	38.33	√
76	Alisol A	√	C_30_H_50_O_5_	[M + FA − H]^−^	535.3640	535.3649	−1.7	40.12	√
77	Cryptotanshinone	√	C_19_H_20_O_3_	[M + H]^+^	297.1485	297.1495	−3.4	40.35	√
78	Torachrysone sulfate	√	C_14_H_14_O_7_S	[M − H]^−^	325.0387	325.0386	0.3	40.42	
79	Tanshinone I		C_18_H_12_O_3_	[M + H]^+^	277.0859	277.0868	−3.2	40.50	√
80	Physcion		C_16_H_12_O_5_	[M + H]^+^	285.0758	285.0767	−3.2	41.37	√
81	Tanshinone IIA		C_19_H_18_O_3_	[M + H]^+^	295.1329	295.1338	−3.0	42.13	√
82	Alisol B,23-acetate		C_32_H_50_O_5_	[M + H]^+^	515.3731	515.3748	−3.3	43.30	√
83	Emodin		C_15_H_10_O_5_	[M − H]^−^	269.0455	269.0456	−0.4	45.37	√

Note: √ means that the compound is compared with the standard information in the database.

**Table 2:** Identification of metabolic components in rat serum after oral administration of EQF.

No.	Component Name	Molecular Formula	Adducts	Expected Mass *m*/*z*	Observed	Error (ppm)	t_R_ (min)
M1	Coclaurine + glucuronidation	C_23_H_27_NO_9_	[M + H]^+^	462.1759	462.1758	0.2	8.56
M2	Nuciferine + didemethylation + glucuronidation	C_23_H_25_NO_8_	[M + H]^+^	444.1653	444.1651	0.5	9.44
M3	Catechin + glucuronidation	C_21_H_22_O_12_	[M − H]^−^	465.1039	465.1038	0.2	9.74
M4	Armepavine + glucuronidation	C_25_H_31_NO_9_	[M + H]^+^	490.2072	490.2069	0.6	10.66
M5	Nuciferine + didemethylation + glucuronidation	C_23_H_25_NO_8_	[M + H]^+^	444.1653	444.1653	0.0	10.93
M6	Coclaurine + glucuronidation	C_23_H_27_NO_9_	[M + H]^+^	462.1759	462.1759	0.0	10.97
M7	Nuciferine + demethylation + glucuronidation	C_24_H_27_NO_8_	[M + H]^+^	458.1809	458.1813	−0.9	11.64
M8	Puerarin + glucuronidation	C_27_H_28_O_15_	[M − H]^−^	591.1355	591.1359	−0.7	12.06
M9	Coclaurine + glucuronidation	C_23_H_27_NO_9_	[M + H]^+^	462.1759	462.1761	−0.4	12.35
M10	Catechin + methylation + glucuronidation	C_22_H_24_O_12_	[M − H]^−^	479.1195	479.1190	1.0	13.01
M11	Liquiritin + glucuronidation	C_27_H_30_O_15_	[M − H]^−^	593.1512	593.1516	−0.7	13.55
M12	Polydatin + glucuronidation	C_26_H_30_O_14_	[M − H]^−^	565.1563	565.1570	−1.2	13.76
M13	Daidzin + sulfation	C_21_H_20_O_12_S	[M − H]^−^	495.0603	495.0603	0.0	15.02
M14	Puerarin + hydroxylation + sulfation	C_21_H_20_O_13_S	[M − H]^−^	511.0552	511.0554	−0.4	15.05
M15	Daidzein + glucuronidation	C_21_H_18_O_10_	[M − H]^−^	429.0827	429.0827	0.0	16.59
M16	Quercetin + diglucuronidation	C_27_H_26_O_19_	[M − H]^−^	653.0996	653.1000	−0.6	16.61
M17	Nuciferine + didemethylation + sulfation	C_17_H_17_NO_5_S	[M + H]^+^	348.0900	348.0901	−0.3	16.64
M18	Daidzin + sulfation	C_21_H_20_O_12_S	[M − H]^−^	495.0603	495.0608	−1.0	17.03
M19	3′-Methoxypuerarin + sulfation	C_22_H_22_O_13_S	[M − H]^−^	525.0710	525.0715	−1.0	17.05
M20	Daidzein + sulfation + glucuronidation	C_21_H_18_O_13_S	[M − H]^−^	509.0395	509.0400	−1.0	17.10
M21	Physcion + glucuronidation + sulfation	C_22_H_20_O_14_S	[M − H]^−^	539.0501	539.0504	−0.6	17.34
M22	Genistein + methylation + glucuronidation	C_22_H_20_O_11_	[M − H]^−^	459.0933	459.0934	−0.2	17.58
M23	Quercetin + diglucuronidation	C_27_H_26_O_19_	[M − H]^−^	653.0996	653.0997	−0.2	17.77
M24	Catechin + methylation + sulfation	C_16_H_16_O_9_S	[M − H]^−^	383.0442	383.0435	1.8	18.03
M25	Liquiritigenin + glucuronidation	C_21_H_20_O_10_	[M − H]^−^	431.0984	431.0978	1.4	18.54
M26	Quercetin + diglucuronidation	C_27_H_26_O_19_	[M − H]^−^	653.0996	653.0990	0.9	18.74
M27	Liquiritigenin + glucuronidation	C_21_H_20_O_10_	[M − H]^−^	431.0984	431.0979	1.2	19.12
M28	Quercetin + diglucuronidation	C_27_H_26_O_19_	[M − H]^−^	653.0996	653.0997	−0.2	19.64
M29	Nuciferine + demethylation	C_18_H_19_NO_2_	[M + H]^+^	282.1489	282.1490	−0.4	19.69
M30	Catechin + methylation + sulfation	C_16_H_16_O_9_S	[M − H]^−^	383.0442	383.0438	1.0	19.77
M31	Genistein + methylation + glucuronidation	C_22_H_20_O_11_	[M − H]^−^	459.0933	459.0932	0.2	19.90
M32	Puerarin + methylation	C_22_H_22_O_9_	[M − H]^−^	429.1190	429.1188	0.5	20.21
M33	Rhein + glucuronidation	C_21_H_16_O_12_	[M − H]^−^	459.0569	459.0567	0.4	20.23
M34	Liquiritigenin + glucuronidation + sulfation	C_21_H_20_O_13_S	[M − H]^−^	511.0552	511.0550	0.4	20.66
M35	Emodin + diglucuronidation	C_27_H_26_O_17_	[M − H]^−^	621.1097	621.1099	−0.3	21.39
M36	Genistein + methylation + glucuronidation	C_22_H_20_O_11_	[M − H]^−^	459.0933	459.0931	0.4	21.82
M37	Daidzin + glucuronidation	C_27_H_28_O_15_	[M − H]^−^	591.1360	591.1357	0.5	21.90
M38	Emodin + sulfation + glucuronidation	C_21_H_18_O_14_S	[M − H]^−^	525.0345	525.0347	−0.4	22.14
M39	Rosmarinic acid + methylation + glucuronidation	C_25_H_26_O_14_	[M − H]^−^	549.1245	549.1256	−2.0	22.17
M40	Morin + sulfation + glucuronidation	C_21_H_18_O_16_S	[M − H]^−^	557.0243	557.0246	−0.5	22.36
M41	Emodin + sulfation + glucuronidation	C_21_H_18_O_14_S	[M − H]^−^	525.0345	525.0346	−0.2	22.44
M42	Quercetin + methylation + glucuronidation	C_22_H_20_O_13_	[M − H]^−^	491.0831	491.0831	0.0	22.77
M43	Quercetin + methylation + glucuronidation + sulfation	C_22_H_20_O_16_S	[M − H]^−^	571.0399	571.0401	−0.4	23.19
M44	Emodin + diglucuronidation	C_27_H_26_O_17_	[M − H]^−^	621.1097	621.1100	−0.5	23.28
M45	Rosmarinicacid + dimethylation + glucuronidation	C_26_H_28_O_14_	[M − H]^−^	563.1406	563.1406	0.0	23.48
M46	TanshinoneI + dihydroxylation + glucuronidation	C_24_H_24_O_11_	[M + H]^+^	489.1391	489.1400	−1.8	23.90
M47	Formononetin + hydroxylation	C_16_H_12_O_5_	[M + H]^+^	285.0758	285.0761	−1.1	24.23
M48	Rosmarinicacid + dimethylation + glucuronidation	C_26_H_28_O_14_	[M − H]^−^	563.1406	563.1409	−0.5	24.28
M49	TanshinoneI + dihydroxylation + glucuronidation	C_24_H_24_O_11_	[M + H]^+^	489.1391	489.1396	−1.0	24.97
M50	Physcion + diglucuronidation	C_28_H_28_O_17_	[M − H]^−^	635.1254	635.1250	0.6	25.05
M51	Daidzein + hydrogenation + glucuronidation	C_21_H_20_O_10_	[M − H]^−^	431.0984	431.0982	0.5	25.49
M52	Physcion + sulfation	C_16_H_12_O_8_S	[M − H]^−^	363.0180	363.0175	1.4	25.94
M53	6-Methylrhein + glucuronidation	C_22_H_18_O_12_	[M − H]^−^	473.0725	473.0724	0.2	26.18
M54	Morin + methylation + glucuronidation	C_22_H_20_O_13_	[M − H]^−^	491.0831	491.0831	0.0	26.42
M55	Rhein + sulfation	C_15_H_8_O_9_S	[M − H]^−^	362.9816	362.9814	0.6	27.15
M56	Daidzein + hydrogenation + sulfation	C_15_H_12_O_7_S	[M − H]^−^	335.0231	335.0221	3.0	27.27
M57	Liquiritigenin + methylation + glucuronidation	C_22_H_22_O_10_	[M − H]^−^	445.1140	445.1127	2.9	27.41
M58	Emodin + glucuronidation	C_21_H_18_O_11_	[M − H]^−^	445.0776	445.0775	0.2	27.73
M59	Daidzein + disulfation	C_15_H_10_O_10_S_2_	[M − H]^−^	412.9643	412.9639	1.0	27.93
M60	Lithospermic acid + dimethylation	C_29_H_26_O_12_	[M − H]^−^	565.1352	565.1357	−0.9	28.29
M61	Cryptotanshinone + trihydroxylation	C_19_H_20_O_6_	[M + H]^+^	345.1333	345.1339	−1.7	28.59
M62	Quercetin + dimethylation + glucuronidation	C_23_H_22_O_13_	[M − H]^−^	505.0988	505.0990	−0.4	28.65
M63	Lithospermic acid + dimethylation	C_29_H_26_O_12_	[M − H]^−^	565.1352	565.1355	−0.5	28.68
M64	Quercetin + dimethylation + glucuronidation	C_23_H_22_O_13_	[M − H]^−^	505.0988	505.0991	−0.6	29.36
M65	Cryptotanshinone + Hydroxylation	C_19_H_20_O_4_	[M + H]^+^	313.1434	313.1439	−1.6	29.80
M66	Physcion + glucuronidation	C_22_H_20_O_11_	[M − H]^−^	459.0933	459.0937	−0.9	30.09
M67	Emodin + glucuronidation	C_21_H_18_O_11_	[M − H]^−^	445.0776	445.0778	−0.4	30.36
M68	Rosmarinic acid + dimethylation + sulfation	C_20_H_20_O_11_S	[M − H]^−^	467.0654	467.0636	3.9	30.60
M69	Physcion + glucuronidation	C_22_H_20_O_11_	[M − H]^−^	459.0933	459.0934	−0.2	30.64
M70	Tanshinone I + hydroxylation	C_18_H_12_O_4_	[M + H]^+^	293.0808	293.0817	−3.1	32.25
M71	Daidzein + hydrogenation + sulfation	C_15_H_12_O_7_S	[M − H]^−^	335.0231	335.0230	0.3	34.89
M72	Tanshinone IIA + hydrogenation	C_19_H_20_O_3_	[M + H]^+^	297.1485	297.1491	−2.0	36.81
M73	Cryptotanshinone + hydroxylation + hydrogenation	C_19_H_22_O_4_	[M + H]^+^	315.1591	315.1599	−2.5	38.49
M74	Emodin + sulfation	C_15_H_10_O_8_S	[M − H]^−^	349.0024	349.0021	0.9	44.39
M75	Emodin + sulfation	C_15_H_10_O_8_S	[M − H]^−^	349.0024	349.0022	0.6	44.70

## Data Availability

The complete mass-spectrometry raw files are available from the corresponding author upon reasonable request due to very large file sizes and proprietary formats. A de-identified minimal dataset that contains all essential data necessary to support the central findings and allows others to verify the results has been uploaded.

## Supplementary Materials

The following supporting information can be downloaded at https://www.mdpi.com/article/10.3390/nu17243849/s1, Figure S1: Chemical structures of the components of EQF identified by UPLC-Q-TOF-MS. (A) Flavonoids. (B) Quinones. (C) Triterpenoid saponins. (D) Triterpenoids. (E) Alkaloids. (F) Phenol; Table S1: Primer sequence for quantitative real-time PCR; File S1: Lipidomics raw; File S2: Metabolomics raw.

## Abbreviations

The following abbreviations are used in this manuscript:

AKT	Protein kinase B
ALT	Alanine aminotransferase
AST	Aspartate aminotransferase
BCA	Bicinchoninic acid
BCL-2	B-cell lymphoma 2
CASP3	Caspase3
EQF	Erhuang Quzhi Formula
ESR1	Estrogen receptor 1
FC	Fold change
GLU	Glucose
H&E	Hematoxylin and eosin
HDL-C	High-density lipoprotein cholesterol
HFD	High-fat diet
LDL-C	Low-density lipoprotein cholesterol
MAPK	Mitogen-activated protein kinase
MMP9	Matrix metalloproteinase 9
NAFLD	Non-alcoholic fatty liver disease
NASH	Non-alcoholic steatohepatitis
ND	Normal diet
OPLS-DA	Orthogonal projections to latent structures–discriminant analysis
PCA	Principal component analysis
PPI	Protein–protein interaction
PVDF	Polyvinylidene fluoride
RT-qPCR	Real-time quantitative polymerase chain reaction
SDS	Sodium dodecyl sulfate
STAT3	Signal transducer and activator of transcription 3
TC	Total cholesterol
TCM	Traditional Chinese medicine
TG	Triglyceride
TNF	Tumor necrosis factor
UCP1	Uncoupling protein 1
UPLC-Q-TOF-MS	Ultra-performance liquid chromatography coupled with quadrupole time-of-flight mass spectrometry
VIP	Variable importance in projection
VLDL	Very-low-density lipoprotein

## References

[1] Eslam M., Sanyal A.J., George J., International Consensus Panel (2020). MAFLD: A Consensus-Driven Proposed Nomenclature for Metabolic Associated Fatty Liver Disease. Gastroenterology.

[2] Saiman Y., Duarte-Rojo A., Rinella M.E. (2022). Fatty Liver Disease: Diagnosis and Stratification. Annu. Rev. Med..

[3] Cotter T.G., Rinella M. (2020). Nonalcoholic Fatty Liver Disease 2020: The State of the Disease. Gastroenterology.

[4] Polyzos S.A., Kountouras J., Mantzoros C.S. (2019). Obesity and nonalcoholic fatty liver disease: From pathophysiology to therapeutics. Metabolism.

[5] Tilg H., Effenberger M. (2020). From NAFLD to MAFLD: When pathophysiology succeeds. Nat. Rev. Gastroenterol. Hepatol..

[6] Neuschwander-Tetri B.A. (2017). Non-alcoholic fatty liver disease. BMC Med..

[7] Eslam M., Ratziu V., George J. (2021). Yet more evidence that MAFLD is more than a name change. J. Hepatol..

[8] Tilg H., Adolph T.E., Moschen A.R. (2021). Multiple Parallel Hits Hypothesis in Nonalcoholic Fatty Liver Disease: Revisited After a Decade. Hepatology.

[9] Singh S.P., Anirvan P., Reddy K.R., Conjeevaram H.S., Marchesini G., Rinella M.E., Madan K., Petroni M.L., Al-Mahtab M., Caldwell S.H. (2021). Non-alcoholic fatty liver disease: Not time for an obituary just yet!. J. Hepatol..

[10] Polyzos S.A., Kechagias S., Tsochatzis E.A. (2021). Review article: Non-alcoholic fatty liver disease and cardiovascular diseases: Associations and treatment considerations. Aliment. Pharmacol. Ther..

[11] Yuan J.Q. (2018). Yuan Jinqi’s Medical Essays.

[12] Li S., Ma Y., Chen W. (2023). Active ingredients of Erhuang Quzhi Granules for treating non-alcoholic fatty liver disease based on the NF-κB/NLRP3 pathway. Fitoterapia.

[13] Shiha G., Mousa N. (2021). Non-alcoholic steatohepatitis or metabolic-associated fatty liver: Time to change. Hepatobiliary Surg. Nutr..

[14] Li S., Wu X., Ma Y., Zhang H., Chen W. (2023). Prediction and verification of the active ingredients and potential targets of Erhuang Quzhi Granules on non-alcoholic fatty liver disease based on network pharmacology. J. Ethnopharmacol..

[15] Pan P., Chen W., Wu X., Li C., Gao Y., Qin D. (2024). Active Targets and Potential Mechanisms of Erhuang Quzhi Formula in Treating NAFLD: Network Analysis and Experimental Assessment. Cell Biochem. Biophys..

[16] Tang Y.P., Xu D.Q., Yue S.J., Chen Y.Y., Fu R.J., Bai X. (2022). Modern research thoughts and methods on bio-active components of TCM formulae. Chin. J. Nat. Med..

[17] Wang X.J., Ren J.L., Zhang A.H., Sun H., Yan G.L., Han Y., Liu L. (2019). Novel applications of mass spectrometry-based metabolomics in herbal medicines and its active ingredients: Current evidence. Mass Spectrom. Rev..

[18] Han Y., Sun H., Zhang A., Yan G., Wang X.J. (2020). Chinmedomics, a new strategy for evaluating the therapeutic efficacy of herbal medicines. Pharmacol. Ther..

[19] Zhao X., Su H., Chen H., Tang X., Li W., Huang A., Fang G., Chen Q., Luo Y., Pang Y. (2024). Integrated serum pharmacochemistry and network pharmacology to explore the mechanism of Yi-Shan-Hong formula in alleviating chronic liver injury. Phytomedicine.

[20] Wu Z., Bagarolo G.I., Thoroe-Boveleth S., Jankowski J. (2020). “Lipidomics”: Mass spectrometric and chemometric analyses of lipids. Adv. Drug Deliv. Rev..

[21] Musso G., Saba F., Cassader M., Gambino R. (2023). Lipidomics in pathogenesis, progression and treatment of nonalcoholic steatohepatitis (NASH): Recent advances. Prog. Lipid Res..

[22] Huang J., Sigon G., Mullish B.H., Wang D., Sharma R., Manousou P., Forlano R. (2023). Applying Lipidomics to Non-Alcoholic Fatty Liver Disease: A Clinical Perspective. Nutrients.

[23] Shen S., Zhan C., Yang C., Fernie A.R., Luo J. (2023). Metabolomics-centered mining of plant metabolic diversity and function: Past decade and future perspectives. Mol. Plant.

[24] Wang R., Li B., Lam S.M., Shui G. (2020). Integration of lipidomics and metabolomics for in-depth understanding of cellular mechanism and disease progression. J. Genet. Genom..

[25] Khanna R.K., Catanese S., Emond P., Corcia P., Blasco H., Pisella P.J. (2022). Metabolomics and lipidomics approaches in human tears: A systematic review. Surv. Ophthalmol..

[26] Perakakis N., Stefanakis K., Mantzoros C.S. (2020). The role of omics in the pathophysiology, diagnosis and treatment of non-alcoholic fatty liver disease. Metabolism.

[27] Ren S., Gao Y., Wang J., Feng J., Li J., Xiang T., Xuan R., Zhou Y. (2025). Yinchenhao decoction and its active compound rhein ameliorate intrahepatic cholestasis of pregnancy in mice via modulation of intestinal flora. Phytomedicine.

[28] Li B., Xiao Q., Zhao H., Zhang J., Yang C., Zou Y., Zhang B., Liu J., Sun H., Liu H. (2024). Schisanhenol ameliorates non-alcoholic fatty liver disease via inhibiting miR-802 activation of AMPK-mediated modulation of hepatic lipid metabolism. Acta Pharm. Sin. B.

[29] Zhang X., Zhang J., Zhou Z., Xiong P., Cheng L., Ma J., Wen Y., Shen T., He X., Wang L. (2024). Integrated network pharmacology, metabolomics, and transcriptomics of Huanglian-Hongqu herb pair in non-alcoholic fatty liver disease. J. Ethnopharmacol..

[30] Targher G., Byrne C.D., Tilg H. (2020). NAFLD and increased risk of cardiovascular disease: Clinical associations, pathophysiological mechanisms and pharmacological implications. Gut.

[31] Younossi Z.M. (2019). Non-alcoholic fatty liver disease—A global public health perspective. J. Hepatol..

[32] Paternostro R., Trauner M. (2022). Current treatment of non-alcoholic fatty liver disease. J. Intern. Med..

[33] Safari Z., Gerard P. (2019). The links between the gut microbiome and non-alcoholic fatty liver disease (NAFLD). Cell. Mol. Life Sci..

[34] Yan T., Yan N., Wang P., Xia Y., Hao H., Wang G., Gonzalez F.J. (2020). Herbal drug discovery for the treatment of nonalcoholic fatty liver disease. Acta Pharm. Sin. B.

[35] Zhao Y., Shen X., Li N., Huang W., Li X., Ye Q., Ruan Y., Li R., Zhu H., Xu L. (2025). Qidong Huoxue decoction protects against acute lung injury by promoting SIRT1-mediated p300 deacetylation. Phytomedicine.

[36] Xia W., Liu B., Tang S., Yasir M., Khan I. (2022). The science behind TCM and Gut microbiota interaction-their combinatorial approach holds promising therapeutic applications. Front. Cell. Infect. Microbiol..

[37] Huyghe J., Priem D., Bertrand M.J.M. (2023). Cell death checkpoints in the TNF pathway. Trends Immunol..

[38] Tiegs G., Horst A.K. (2022). TNF in the liver: Targeting a central player in inflammation. Semin. Immunopathol..

[39] Zhao Y., Zhang T., Liang Y., Xie X., Pan H., Cao M., Wang S., Wu D., Wang J., Wang C. (2024). Combination of aloe emodin, emodin, and rhein from Aloe with EDTA sensitizes the resistant Acinetobacter baumannii to polymyxins. Front. Cell. Infect. Microbiol..

[40] Hu Y., Yang L., Lai Y. (2023). Recent findings regarding the synergistic effects of emodin and its analogs with other bioactive compounds: Insights into new mechanisms. Biomed. Pharmacother..

[41] Li X., Peng Y., Liu H., Xu Y., Wang X., Zhang C., Ma X. (2020). Comparative studies on the interaction of nine flavonoids with trypsin. Spectrochim. Acta A Mol. Biomol. Spectrosc..

[42] Cheng K.W., Shi J., Huang C., Tan H.Y., Ning Z., Lyu C., Xu Y., Mok H.L., Zhai L., Xiang L. (2024). Integrated metabolomics and serum-feces pharmacochemistry-based network pharmacology to reveal the mechanisms of an herbal prescription against ulcerative colitis. Comput. Biol. Med..

[43] Zhu Z., Feng Y.D., Zou Y.L., Xiao Y.H., Wu J.J., Yang Y.R., Jiang X.X., Wang L., Xu W. (2024). Integrating serum pharmacochemistry, network pharmacology and untargeted metabolomics strategies to reveal the material basis and mechanism of action of Feining keli in the treatment of chronic bronchitis. J. Ethnopharmacol..

[44] Yuan X., He X., Shu Q., Gao Y., Chen Y., Xu J., Zhang Y., Cao G. (2025). Integrating serum pharmacochemistry, network pharmacology, and metabolomics to explore the protective mechanism of Hua-Feng-Dan in ischemic stroke. Phytomedicine.

[45] Liu Z., Tian Z., Zhao D., Liang Y., Dai S., Liu M., Hou S., Dong X., Zhaxinima, Yang Y. (2022). Effects of Coenzyme Q10 Supplementation on Lipid Profiles in Adults: A Meta-analysis of Randomized Controlled Trials. J. Clin. Endocrinol. Metab..

[46] Jiang J., Kao T.C., Hu S., Li Y., Feng W., Guo X., Zeng J., Ma X. (2023). Protective role of baicalin in the dynamic progression of lung injury to idiopathic pulmonary fibrosis: A meta-analysis. Phytomedicine.

[47] Wan Y., Wang J., Xu J.F., Tang F., Chen L., Tan Y.Z., Rao C.L., Ao H., Peng C. (2021). Panax ginseng and its ginsenosides: Potential candidates for the prevention and treatment of chemotherapy-induced side effects. J. Ginseng Res..

[48] Kivimaki M., Bartolomucci A., Kawachi I. (2023). The multiple roles of life stress in metabolic disorders. Nat. Rev. Endocrinol..

[49] Wilkerson J.L., Tatum S.M., Holland W.L., Summers S.A. (2024). Ceramides are fuel gauges on the drive to cardiometabolic disease. Physiol. Rev..

[50] Chaurasia B., Summers S.A. (2021). Ceramides in Metabolism: Key Lipotoxic Players. Annu. Rev. Physiol..

[51] Apostolopoulou M., Gordillo R., Koliaki C., Gancheva S., Jelenik T., De Filippo E., Herder C., Markgraf D., Jankowiak F., Esposito I. (2018). Specific Hepatic Sphingolipids Relate to Insulin Resistance, Oxidative Stress, and Inflammation in Nonalcoholic Steatohepatitis. Diabetes Care.

[52] Lu Z., Li Y., Chowdhury N., Yu H., Syn W.K., Lopes-Virella M., Yilmaz Ö., Huang Y. (2023). The Presence of Periodontitis Exacerbates Non-Alcoholic Fatty Liver Disease via Sphingolipid Metabolism-Associated Insulin Resistance and Hepatic Inflammation in Mice with Metabolic Syndrome. Int. J. Mol. Sci..

[53] Powers M.J., Trent M.S. (2019). Intermembrane transport: Glycerophospholipid homeostasis of the Gram-negative cell envelope. Proc. Natl. Acad. Sci. USA.

[54] Varandas P., Cobb A.J.A., Segundo M.A., Silva E.M.P. (2020). Emergent Glycerophospholipid Fluorescent Probes: Synthesis and Applications. Bioconjug. Chem..

[55] Jiang X., Fulte S., Deng F., Chen S., Xie Y., Chao X., He X.C., Zhang Y., Li T., Li F. (2022). Lack of VMP1 impairs hepatic lipoprotein secretion and promotes non-alcoholic steatohepatitis. J. Hepatol..

[56] Presa N., Clugston R.D., Lingrell S., Kelly S.E., Merrill A.H., Jana S., Kassiri Z., Gómez-Muñoz A., Vance D.E., Jacobs R.L. (2019). Vitamin E alleviates non-alcoholic fatty liver disease in phosphatidylethanolamine N-methyltransferase deficient mice. Biochim. Biophys. Acta Mol. Basis Dis..

[57] Fiorucci S., Marchiano S., Urbani G., Di Giorgio C., Distrutti E., Zampella A., Biagioli M. (2024). Immunology of bile acids regulated receptors. Prog. Lipid Res..

