# Supplementary material for: A Multi-Omics Study Reveals the Active Components and Therapeutic Mechanism of Erhuang Quzhi Formula for Non-Alcoholic Fatty Liver Disease
Source: Nutrients. 2025 Dec 10;17(24):3849. doi: 10.3390/nu17243849 (PMC12736208; doi:10.3390/nu17243849)

Table S1. Primer sequence for Quantitative real-time PCR.

Gene Name	Forward Primer (5′-3′)	Reverse Primer (5′-3′)
Bcl-2	GACTGAGTACCTGAACCGGC	AGTTCCACAAAGGCATCCCAG
Stat3	GTAGACAGGGAGGGGGAACC	AGTCAGGGGTCTCGACTGTCT
Caspase3	GAGCTTGGAACGGTACGCTA	GCGAGATGACATTCCAGTGC
Esr1	TGAGGATGTTAGGCTTCGTCT	TCCACTCCCACAATGCACAC
Tnf	TTGAACCAGCAGGGTGGC	CGAGTTACAGACTGGCTCCC

Figure S1. Chemical structures of the components of EQF identified by UPLC-Q-TOF-MS. (**A**) Flavonoids. (**B**) Quinones. (**C**) Triterpenoid saponins. (**D**) Triterpenoids. (**E**) Alkaloids. (**F**) Phenol.

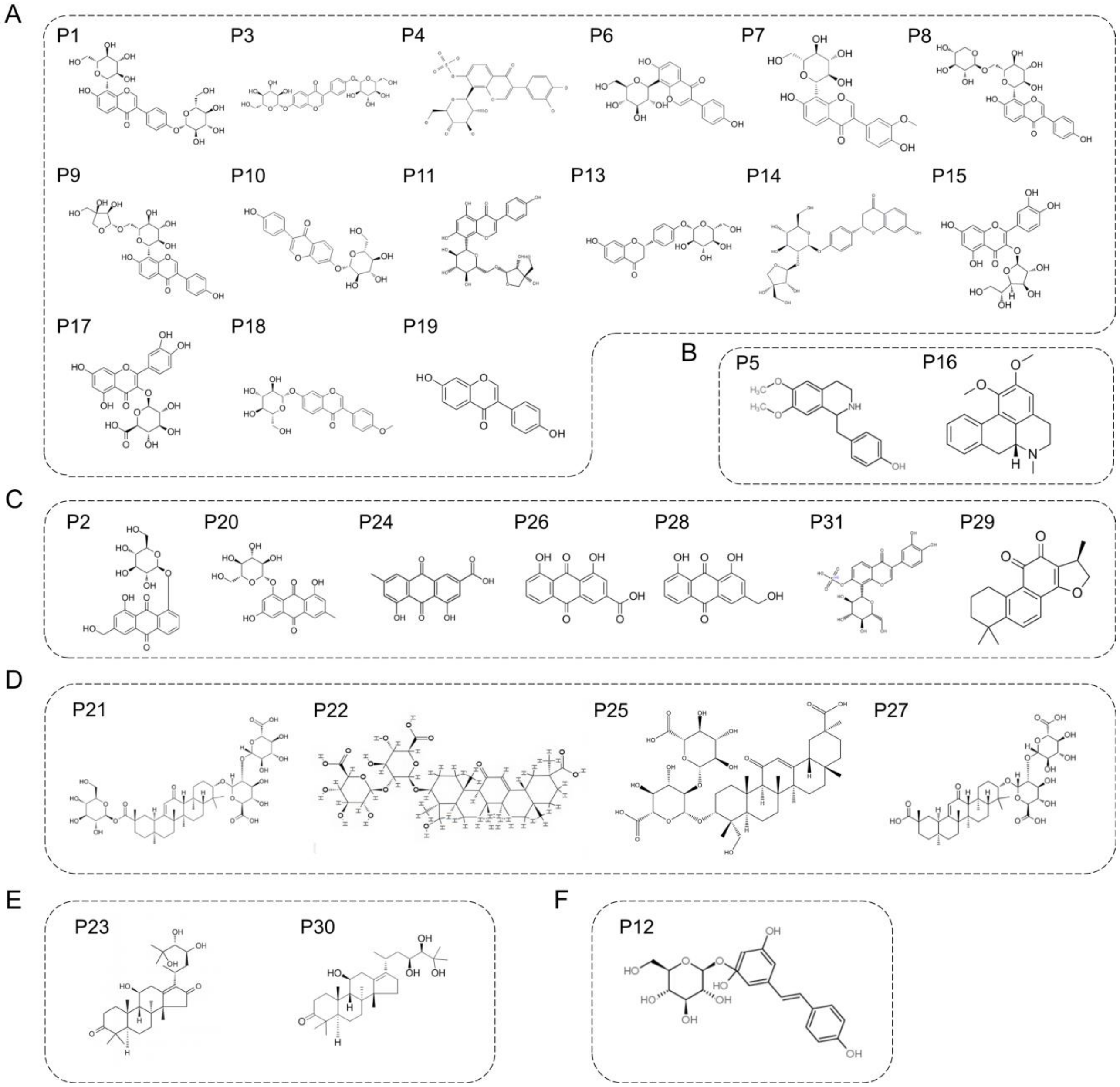

Supplement: Supplementary file 1 [file nutrients-17-03849-s001.zip › Supplementary figure and table.pdf]
